# Hypoxia‐Mimicking Mediated Macrophage‐Elimination of Erythrocytes Promotes Bone Regeneration via Regulating Integrin α_v_β_3_/Fe^2+^‐Glycolysis‐Inflammation

**DOI:** 10.1002/advs.202403921

**Published:** 2024-10-01

**Authors:** Yong Ao, Yuanlong Guo, Yingye Zhang, Lv Xie, Ruidi Xia, Jieyun Xu, Mengru Shi, Xiaomeng Gao, Xiaoran Yu, Zetao Chen

**Affiliations:** ^1^ Hospital of Stomatology Guanghua School of Stomatology Sun Yat‐sen University Guangdong Research Center for Dental and Cranial Rehabilitation and Material Engineering Guangzhou 510055 China

**Keywords:** bone regeneration, erythrocyte clearance, hypoxia‐mimicking, inflammatory responses, macrophage phagocytosis

## Abstract

Erythrocytes are the dominant component of a blood clot in terms of volume and number. However, longstanding compacted erythrocytes in blood clots form a physical barrier and make fibrin mesh more anti‐fibrinolytic, thus impeding infiltration of mesenchymal stem cells. The necrosis or lysis of erythrocytes that are not removed timely can also lead to the release of pro‐inflammatory toxic metabolites, interfering with bone regeneration. Proper bio‐elimination of erythrocytes is essential for an undisturbed bone regeneration process. Here, hypoxia‐mimicking is applied to enhance macrophage‐elimination of erythrocytes. The effect of macrophage‐elimination of erythrocytes on the macrophage intracellular reaction, bone regenerative microenvironment, and bone regeneration outcome is investigated. Results show that the hypoxia‐mimicking agent dimethyloxalylglycine successfully enhances erythrophagocytosis by macrophages in a dose‐dependent manner primarily by up‐regulating the expression of integrin α_v_β_3_. Increased phagocytosed erythrocytes then regulate macrophage intracellular Fe^2+^‐glycolysis‐inflammation, creating an improved bone regenerative microenvironment characterized by loose fibrin meshes with down‐regulated local inflammatory responses in vivo, thus effectively promoting early osteogenesis and ultimate bone generation. Modulating macrophage‐elimination of erythrocytes can be a promising strategy for eradicating erythrocyte‐caused bone regeneration hindrance and offers a new direction for advanced biomaterial development focusing on the bio‐elimination of erythrocytes.

## Graphical abstract

Longstanding compacted erythrocytes impair bone regeneration. This study employs the hypoxia‐mimetic agent DMOG to enhance macrophage‐elimination of erythrocytes by up‐regulating integrin α_v_β_3_. Increased phagocytosed erythrocytes regulate macrophage intracellular Fe^2+^‐glycolysis‐inflammation, creating a favorable bone regenerative microenvironment characterized by loose fibrin meshes with down‐regulated inflammatory responses in vivo. These findings pave the way for advanced biomaterial development focusing on the bio‐elimination of erythrocytes.

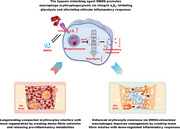

## 1 Introduction

Coagulation is a natural process that forms a blood clot following injury to prevent excessive bleeding.^[^ ^1^ ^]^ The clot forms through the creation of a fibrin network that traps ≈95% erythrocytes, 5% platelets, less than 1% leukocytes,^[^ ^2^ ^]^ and a variety of osteogenic and angiogenic cytokines.^[^ ^3^ ^]^ This clot not only recruits immune cells and mesenchymal stem cells (MSCs) but also acts as a temporary scaffold for these cells, initiating the healing of bone defects^[^ ^4^ ^]^ and playing a crucial role in determining the eventual outcome of the healing process.

As the most abundant component of a blood clot, erythrocytes promote fibrin network forming during coagulation.^[^ ^5^ ^]^ However, upon the clot contraction, compacted erythrocytes form a physical barrier that impedes MSCs infiltration.^[^ ^6^ ^]^ In addition, longstanding erythrocytes interact with the fibrin network and make it denser, interfering with the normal fibrinolysis process,^[^ ^7^ ^]^ which is unfavorable for bone healing.^[^ ^8^ ^]^ Furthermore, necrosis or lysis of erythrocytes can release excess harmful metabolic chemicals including iron, which significantly compromises MSCs viability and triggers excessive inflammatory responses.^[^ ^9^ ^]^ Taken together, longstanding erythrocytes act as a neglected “badass” that reduces the osteogenic capacity of blood clots, thereby impeding natural bone repair. Therefore, “erythrocyte clearance” can be a promising strategy for enhancing bone regeneration.

To effectively remove the overwhelming longstanding erythrocytes, especially in hematoma, mechanical scraping was performed to prevent excessive inflammation and infection, but it resulted in bone nonunion instead.^[^ ^4^ ^]^ The problem lies in the removal of the entire hematoma, which not only eliminates the harmful erythrocytes but also inadvertently removes beneficial elements, including growth factors, as well as osteogenic and angiogenic factors. Therefore, to properly handle erythrocyte clearance, precise bio‐elimination should be developed.

Macrophages are major professional phagocytes that recognize and engulf apoptotic or necrotic cells via several pattern recognition and opsonic receptors expressed in their cell membranes.^[^ ^10^ ^]^ Moreover, macrophages have evolved solid mechanisms for iron ion separation, absorption, storage, utilization, and outflow to avoid iron toxicity,^[^ ^11^ ^]^ providing them with a relatively high tolerance to erythrocytes. These prevent the inflammatory response of macrophages from being easily triggered during erythrophagocytosis, making macrophages the proper candidate for regulating the bio‐elimination of erythrocytes.

Hypoxia has been demonstrated to enhance macrophage phagocytosis of apoptotic neutrophils and latex beads.^[^ ^12^, ^13^ ^]^ Additionally, hypoxia serves as a tissue injury repair signal as well,^[^ ^14^ ^]^ suggesting its potential use in promoting macrophage‐elimination of erythrocytes during bone regeneration. Oxygen deficiency inhibits the activity of the prolyl hydroxylase domain (PHD) enzymes, which prevents the degradation of the hypoxia‐inducible factor (HIF) and promotes its nuclear translocation, thus initiating the hypoxia responses.^[^ ^15^ ^]^ However, direct oxygen deprivation would not only cause harmful oxidative stress,^[^ ^16^ ^]^ but also facilitates the survival and proliferation of anaerobic bacteria,^[^ ^17^ ^]^ resulting in infection and failed bone regeneration. Hypoxia‐mimicking can inhibit PHD activity without oxygen deprivation,^[^ ^18^ ^]^ making it a promising strategy for modulating macrophage‐elimination of erythrocytes. Dimethyloxalylglycine (DMOG) has shown higher specificity and biosafety in inhibiting PHD activity compared to agents like Co^2+^ and desferrioxamine,^[^ ^17^, ^19^ ^]^ making it a suitable candidate to modulate macrophage erythrophagocytosis.

**Figure 1:**
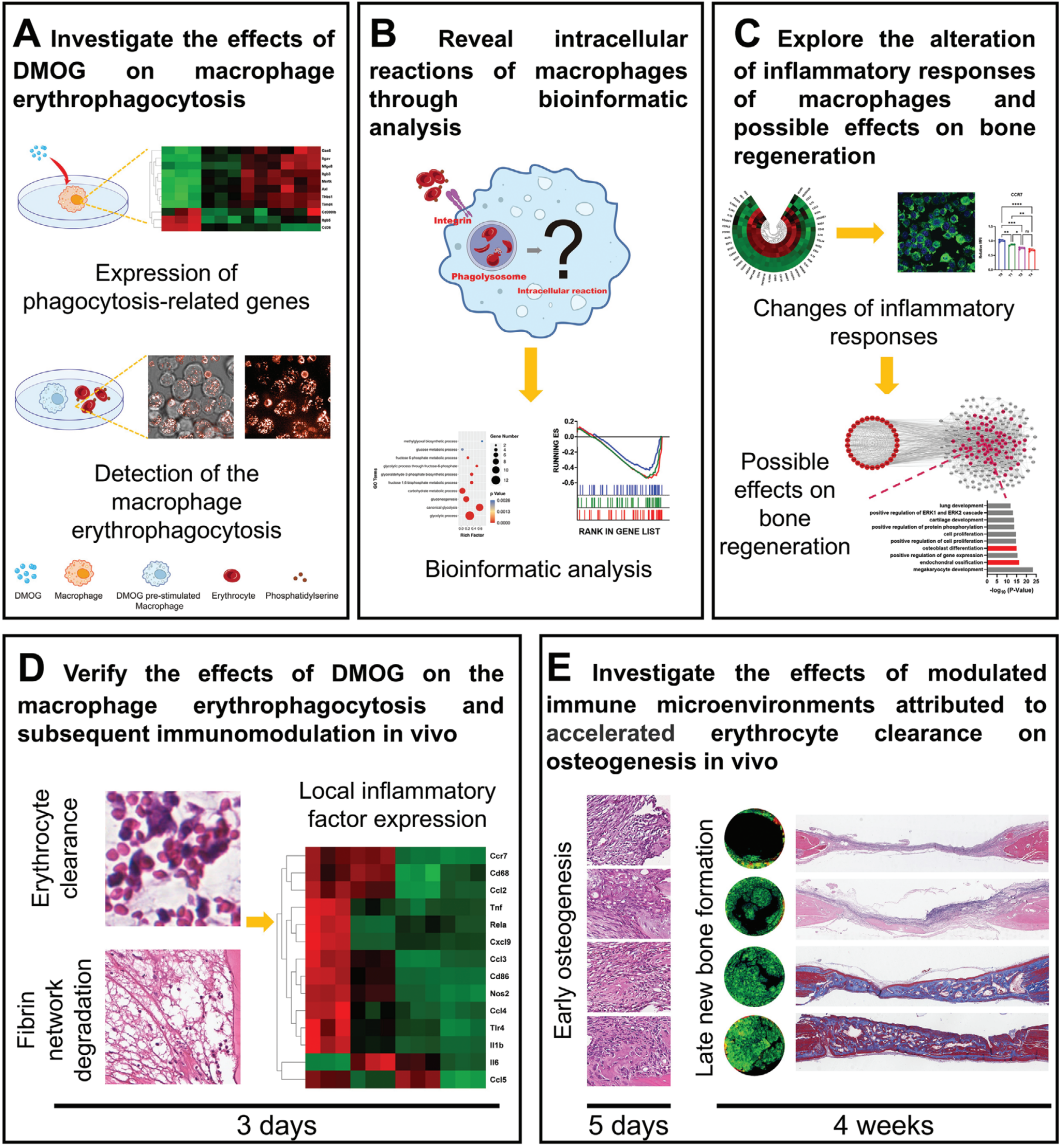
The experimental flow of this study. A) The influence of DMOG on the expression of phagocytosis‐promoting genes was first investigated and then the DMOG pre‐stimulated macrophages were co‐cultured with erythrocytes to detect macrophage erythrophagocytosis. B) Intracellular reactions of macrophages after erythrophagocytosis were revealed by bioinformatic analysis. C) Changes in inflammatory responses were further explored and verified through immunofluorescence staining and RT‐qPCR. The possible effects on bone regeneration were analyzed through bioinformatic analysis. D) Blood clots loaded with DMOG were implanted into rat calvarial defects to verify the effect of DMOG on erythrocyte clearance, fibrin network degradation, and local inflammation in vivo through H&E staining and RT‐qPCR. E) The influence of changed bio‐elimination of erythrocytes on early osteogenesis and late new bone formation was detected through H&E staining, Micro‐CT scanning, and Masson's trichrome staining.

In this study, the dose‐dependent effect of DMOG on erythrophagocytosis of macrophages, and the consequent intracellular reactions and immunological impacts were first investigated in vitro. DMOG‐loaded blood clots were applied to rat calvarial defects to assess the effects of hypoxia‐mimicking on erythrocytes clearance, and the subsequent influence on inflammation and bone regeneration in vivo (**Figure** 1). This study highlighted the significance of the bio‐elimination of erythrocytes during bone regeneration, inspiring the development of advanced biomaterials focusing on the bio‐elimination of erythrocytes to promote bone regeneration. Additionally, this study confirmed that hypoxia‐mimicking can modulate macrophage‐elimination of erythrocytes, offering deeper insights into the function of hypoxia‐mimicking biomaterials.

## 2 Results and Discussion

### 2.1 Macrophage Erythrophagocytosis was Enhanced by the Hypoxia‐Mimicking Agent DMOG in a Dose‐Dependent Manner

**Figure 2:**
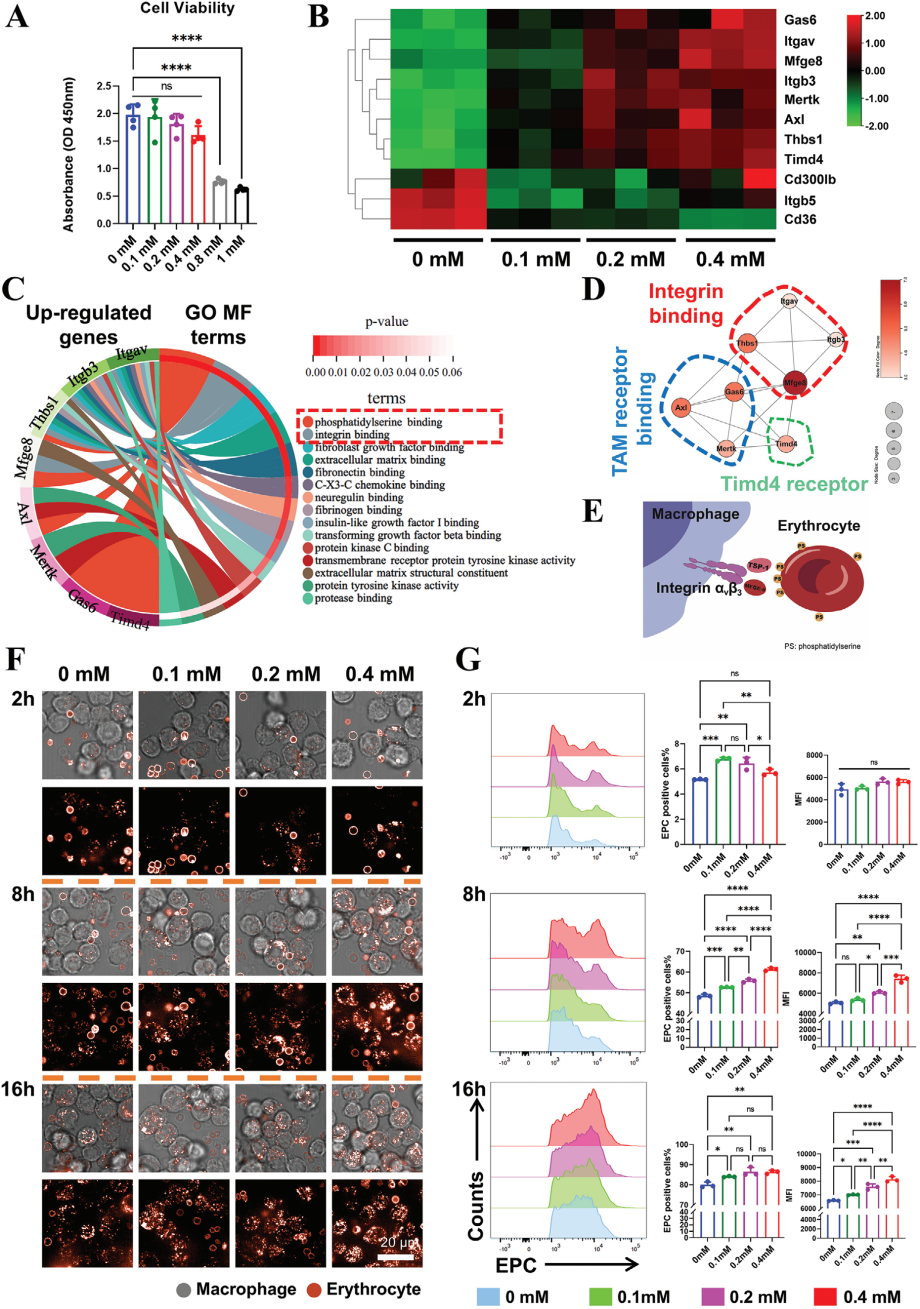
Enhanced erythrophagocytosis of macrophages after stimulation with the hypoxia mimetic agent DMOG in gradient concentrations. A) Cell viability of macrophages after DMOG stimulation (n = 4). B) Gene expression heatmap of typical phagocytosis‐promoting molecules of macrophages after DMOG stimulation (n = 3). C) GO enrichment results and D) Interaction network analysis of the up‐regulated typical phagocytosis‐promoting molecules revealed that DMOG enhanced the EPC of macrophages by regulating recognition between integrin α_v_β_3_ and phosphatidylserine. E) Diagram displays the mechanism by which DMOG pre‐stimulated macrophages phagocytose erythrocytes. F) Representative fluorescence images and G) Flow cytometry results of EPC in macrophages pre‐stimulated with DMOG demonstrated that DMOG effectively promoted the EPC of macrophages (n = 3). PS: phosphatidylserine; EPC: erythrophagocytosis; MFI: mean fluorescence intensity. Data presented as mean ± SD; ^*^ *p *< 0.05, ^**^ *p *< 0.01, ^***^ *p *< 0.001, ^****^ *p *< 0.0001, and ns means not significant by one‐way ANOVA with Tukey's post hoc test.

The safe concentration threshold of DMOG was initially assessed through the CCK8 assay. Results indicated that low concentrations of DMOG (0‐0.4 mm) had no significant impact on macrophage proliferation, whereas higher concentrations (0.8–1.0 mm) inhibited macrophage proliferation (**Figure** **2**A). These findings align with previous reports on DMOG's cytotoxicity toward monocytes and macrophages.^[^ ^20^ ^]^ Subsequently, concentrations of 0–0.4 mm DMOG were applied to a rat calvarial bone defect model to evaluate in vivo systemic toxicity. Both macroscopic and microscopic observations revealed no signs of hyperemia, ischemia, necrosis, or atrophy in rat organs (Figure S1A,B, Supporting Information), indicating no significant systemic toxicity. Consequently, DMOG concentrations of 0, 0.1, 0.2, and 0.4 mm were selected for further experiments.

To investigate the effect of DMOG on macrophage erythrophagocytosis (EPC), the gene expression of EPC‐promoting molecules, including receptors and bridging molecules was first examined by RT‐qPCR. Results showed that DMOG significantly up‐regulated the expression of tyrosine kinase receptor family molecules (AXL/MERTK), TIM4, integrin receptors (integrin α_v_, integrin β_3_), and their bridging molecules (GAS‐6, THBS1, and MFG‐E8) (Figure 2B). TIM4 can directly recognize exposed phosphatidylserine on the erythrocyte surface,^[^ ^21^ ^]^ while AXL/MERTK and integrins can also bind phosphatidylserine through bridging molecules like GAS‐6 and MFG‐E8.^[^ ^22^, ^23^ ^]^ These upregulated genes were further subjected to Gene Ontology (GO) enrichment analysis to elucidate the potential mechanism by which DMOG enhances the phagocytic capacity of macrophages. The analysis revealed that these up‐regulated genes were mainly enriched in phosphatidylserine binding and integrin binding terms (Figure 2C). Protein‐protein interaction analysis revealed that bridging molecules MFG‐E8 and THBS1 are the most correlated, which primarily mediate the binding of integrin α_v_β_3_ to the phosphatidylserine (Figure 2D). Taken together, these findings indicate that DMOG enhances macrophage EPC primarily through the integrin α_v_β_3_‐MFG‐E8/THBS1‐phosphatidylserine recognition (Figure 2E).

To confirm the effect of DMOG on macrophage EPC, macrophages pre‐stimulated with DMOG were co‐cultured with fluorescently labeled, heat‐shocked erythrocytes that externalized phosphatidylserine. After 2 h of co‐culture, erythrocyte engulfment was inconspicuous across all groups (Figure 2F,G). However, after 8 h, confocal images showed that DMOG increased the EPC of macrophages in a dose‐dependent manner, with the most significant effect observed in the 0.4 mm group. Flow cytometry results further indicated that DMOG promoted erythrocyte engulfment by increasing both the proportion of EPC‐positive macrophages and the phagocytic burden per individual macrophage, as reflected by intracellular mean fluorescence intensity (Figure 2F,G). After 16 h, although both DMOG pre‐stimulated and unstimulated groups showed a high proportion of EPC‐positive cells, the intracellular mean fluorescence intensity still exhibited a dose‐dependent increase (Figure 2F,G), indicating that DMOG primarily increased the phagocytic burden of macrophages during the later stages.

In summary, within safe concentration thresholds, DMOG enhanced macrophage erythrophagocytosis by upregulating the expression of phagocytosis‐promoting molecules, particularly integrin α_v_β_3_.

### 2.2 Increased Erythrocyte Engulfment Suppressed the Glycolysis Metabolism of Macrophages

To investigate the effect of increased erythrocyte engulfment on macrophage intracellular reactions, macrophages were pre‐stimulated with 0, 0.1, 0.2, and 0.4 mm DMOG for 24 h and then co‐cultured with heat‐shocked erythrocytes (designated as T0, T1, T2, and T4 groups, respectively). These macrophages were then collected for RNA sequencing (RNA‐seq) and bioinformatic analysis. The principal component analysis result showed that macrophages in the T1, T2, and T4 groups were segregated from those in the T0 group (Figure S2A, Supporting Information), indicating that phagocytosed erythrocytes significantly altered the intracellular reactions of macrophages.

**Figure 3:**
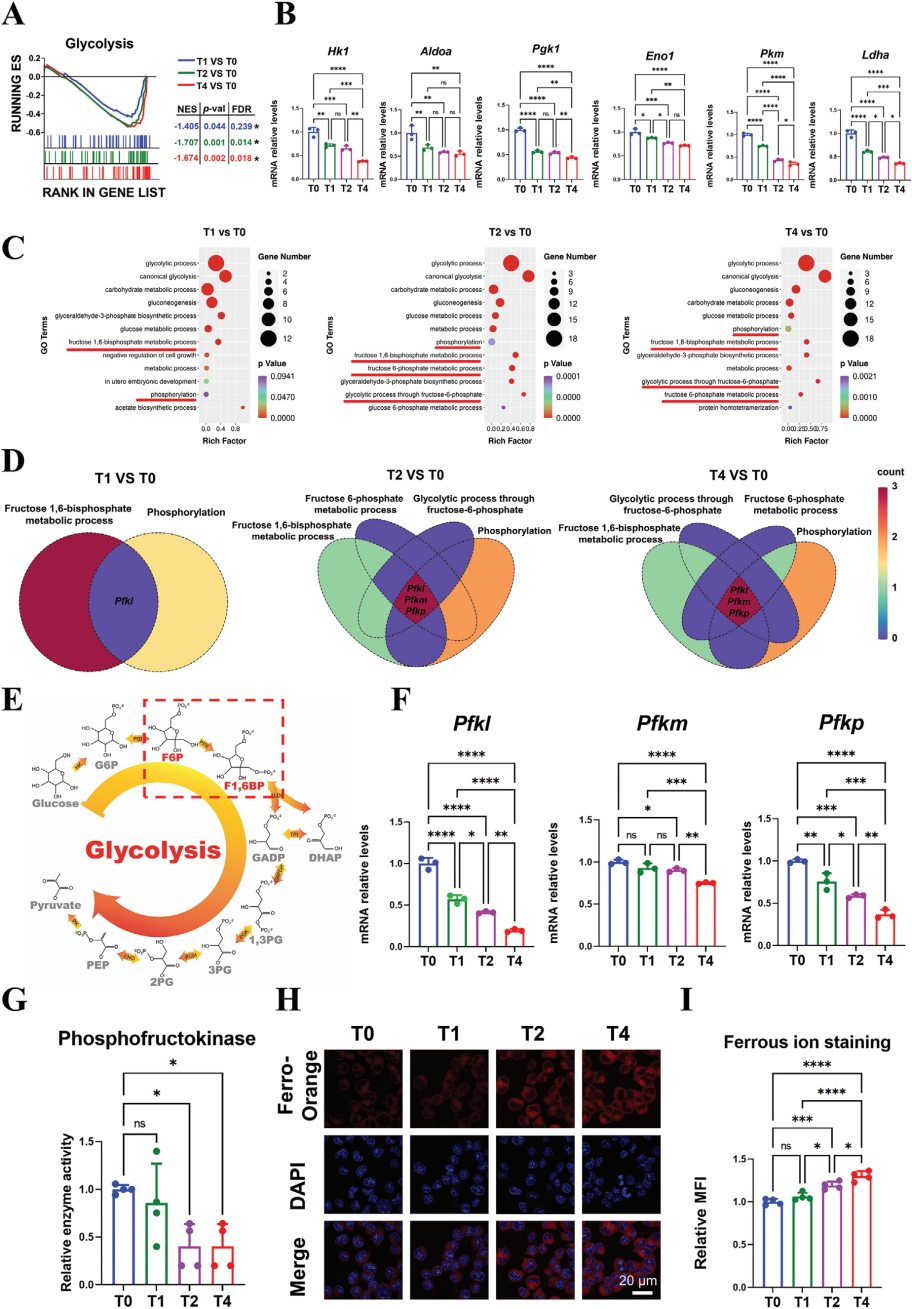
Alternation of intracellular reactions of macrophages after increased erythrocyte engulfment. A) GSEA enrichment results of the glycolysis gene set demonstrated that glycolysis was significantly down‐regulated in T1, T2, and T4 groups (^*^|NES| > 1, *p *<0.05, and FDR < 0.25). B) The gene expression of glycolysis‐related enzymes was investigated by RT‐qPCR (n = 3). C) GO enrichment analysis of the leading genes of glycolysis gene set further revealed the primarily affected processes of glycolysis. D) The Venn diagram analysis showed that *Pfkl*, *Pfkm*, and *Pfkp* were common genes among the selected terms, all encoding phosphofructokinase. E) The schematic diagram of glycolysis process with the primarily affected step marked with a red box. F) RT‐qPCR results showed that the gene expression of *Pfkl*, *Pfkm*, and *Pfkp* was significantly down‐regulated in T1, T2, and T4 groups (n = 3). G) The enzyme activity assay showed decreased enzyme activity of phosphofructokinase after enhanced erythrocyte engulfment (n = 4). H‐I) Representative immunofluorescence images of intracellular Fe^2+^ and the semi‐quantitative statistical analysis results (n = 4). MFI: mean fluorescence intensity. Data presented as mean ± SD; ^*^ *p *< 0.05, ^**^ *p *< 0.01, ^***^ *p *< 0.001, ^****^ *p *< 0.0001, and ns means not significant by one‐way ANOVA with Tukey's post hoc test.

Differential genes in the T1, T2, and T4 groups, compared to the T0 group, were subjected to pathway annotation with the Kyoto Encyclopedia of Genes and Genomes (KEGG). Results showed that cell metabolism‐related pathways accounted for 3/15, 5/15, and 7/15 of the top 15 enriched pathways, respectively (Figure S2B, Supporting Information), indicating that increased erythrocyte engulfment significantly altered macrophage metabolism. The interaction of these metabolism‐related genes was further investigated (Figure S2C, Supporting Information), and the core genes were applied in GO enrichment analysis. Results revealed that glycolysis was the most significant event among the top 10 terms in the T2 and T4 groups (Figure S2D, Supporting Information). Gene set enrichment analysis (GSEA) further demonstrated that the glycolysis pathway was significantly down‐regulated in the T1, T2, and T4 groups (**Figure** **3**A), supported by the expression heatmap of glycolysis‐related genes (Figure S2E, Supporting Information). RT‐qPCR results further verified that the expression of typical glycolytic enzyme genes was down‐regulated in the T1, T2, and T4 groups (Figure 3B), indicating that increased erythrocyte engulfment decreased glycolysis in macrophages.

Glycolysis is a multi‐step biochemical reaction involving 10 enzymes and various intermediate metabolites.^[^ ^24^ ^]^ To identify which step of glycolysis was altered, leading genes of the glycolysis pathway were subjected to GO enrichment analysis. These genes were primarily enriched in terms related to “phosphorylation”, “fructose 1,6‐bisphosphate metabolic process”, “fructose 6‐phosphate metabolic process”, and “glycolytic process through fructose 6‐phosphate” (Figure 3C). Venn diagram analysis revealed that these terms all included *Pfkl*, *Pfkm*, and *Pfkp* (Figure 3D), which encode phosphofructokinase, the key rate‐limiting enzyme that catalyzes the conversion of fructose 6‐phosphate into fructose 1,6‐bisphosphate,^[^ ^25^, ^26^ ^]^ thereby controlling glycolytic flux (Figure 3E). It was found that *Pfkl*, *Pfkm*, and *Pfkp* were gradually down‐regulated in the T1, T2, and T4 groups (Figure S2F, Supporting Information), which was confirmed by RT‐qPCR results (Figure 3F). An enzymatic activity assay further showed that phosphofructokinase activity was significantly decreased in the T2 and T4 groups (Figure 3G). These results indicate that macrophage glycolysis was reduced due to the decreased phosphofructokinase activity, limiting the phosphorylation of fructose 6‐phosphate following enhanced erythrocyte engulfment.

Erythrocytes are a major natural reservoir of iron, which is contained in the heme.^[^ ^27^, ^28^ ^]^ When phagocytosed by macrophages, heme can be degraded into biliverdin, CO, and Fe^2+^.^[^ ^29^ ^]^ It has been reported that Fe^2+^ can inhibit phosphofructokinase activity.^[^ ^30^, ^31^ ^]^ Immunofluorescence images showed that intracellular Fe^2+^ content increased in the T1, T2, and T4 groups (Figure 3H), which was further supported by the semi‐quantitative statistical result (Figure 3I). This increase in Fe^2+^ corresponded with the decreased activity of phosphofructokinase in the T1, T2, and T4 groups. Therefore, it was deduced that phagocytosed erythrocytes reduced phosphofructokinase activity and suppressed glycolysis through Fe^2+^.

In summary, the enhanced erythrocyte engulfment increased intracellular Fe^2+^ content in macrophages, inhibiting phosphofructokinase activity and down‐regulating glycolysis metabolism.

### 2.3 Increased Erythrocyte Engulfment Down‐Regulated Macrophage Inflammatory Responses That Modulate Bone Regenerative Events

Glycolysis is the dominant metabolic pattern of classically activated macrophages (M1).^[^ ^32^, ^33^ ^]^ Additionally, it is widely reported that glycolysis affects macrophage inflammatory responses,^[^ ^34^, ^35^ ^]^ and genes associated with glycolysis are closely correlated with those involved in inflammatory responses (Figure S3A, Supporting Information). Therefore, it was hypothesized that decreased glycolysis would modulate macrophage M1 polarization and inflammatory responses.

**Figure 4:**
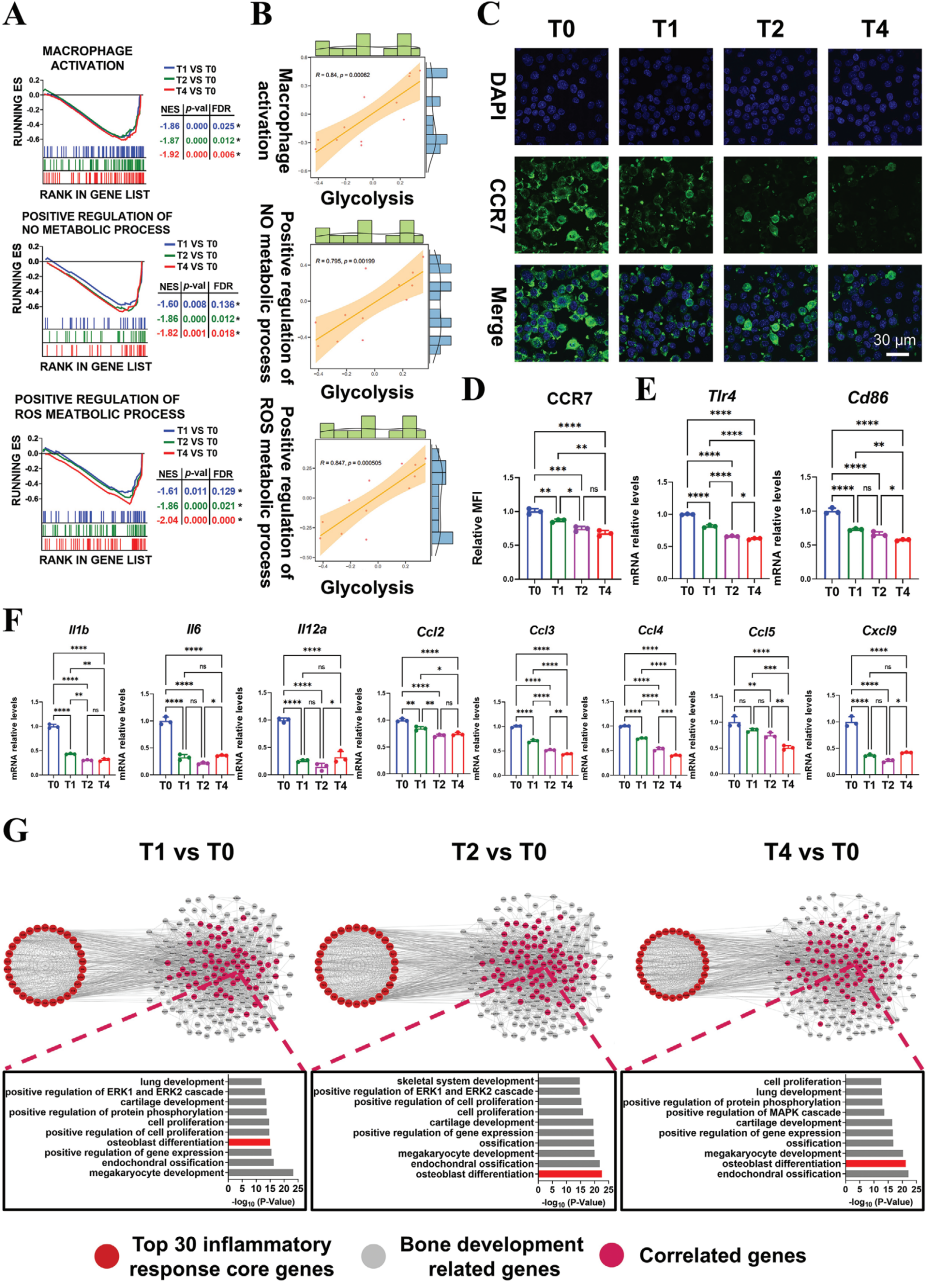
Down‐regulation of macrophage inflammatory responses after increased erythrocyte engulfment and the possible effects on bone regeneration events. A) GSEA enrichment results of macrophage M1 phenotype‐related gene sets (^*^|NES| > 1, *p *<0.05, and FDR < 0.25). B) Spearman's rank correlation analysis of glycolysis and the above three M1 phenotype‐related gene sets. C‐D) Representative immunofluorescence images of CCR7 and the semi‐quantitative statistical analysis results (n = 3). E) RT‐qPCR results of genes of typical M1‐marker TLR4 and CD86 (n = 3). F) RT‐qPCR results of genes of typical inflammation‐related cytokines or chemokines (n = 3). G) Interaction between the top 30 core inflammatory genes and bone development‐related genes, as well as the GO enrichment results of the correlated genes, revealing that the top 30 core inflammatory genes were closely associated with bone regeneration, especially influencing osteoblast differentiation. MFI: mean fluorescence intensity. Data presented as mean ± SD; ^*^ *p *< 0.05, ^**^ *p *< 0.01, ^***^ *p *< 0.001, ^****^ *p *< 0.0001, and ns means not significant by one‐way ANOVA with Tukey's post hoc test.

GSEA results showed that three M1 phenotype‐related terms, including macrophage activation, positive regulation of NO metabolic process, and positive regulation of ROS metabolic process, were significantly down‐regulated in the T1, T2, and T4 groups (**Figure** **4**A). Spearman's rank correlation analysis further revealed a strong positive correlation (R > 0.7) between glycolysis and these three M1 phenotype‐related terms (Figure 4B), indicating that decreased glycolysis contributed to inhibited M1 polarization. Immunofluorescence staining and RT‐qPCR results further showed that the expression of typical M1 phenotype‐related markers (CCR7, TLR4, CD86) was significantly decreased in the T1, T2, and T4 groups (Figure 4C–E). Taken together, these results indicate that increased erythrocyte engulfment‐induced glycolysis suppression down‐regulated macrophage M1 polarization.

Macrophage inflammatory responses after enhanced erythrophagocytosis were evaluated. GSEA results showed that “cytokine production involved in inflammatory responses”, “cytokine‐cytokine receptor interaction”, “chemokine production”, and “chemokine signaling pathway” were all significantly down‐regulated in the T1, T2, and T4 groups (Figure S3B, Supporting Information). Spearman's rank correlation analysis confirmed significant positive correlations between the glycolysis gene set and these inflammatory response‐related terms (Figure S3C, Supporting Information). RT‐qPCR results further showed that the expression of typical inflammatory cytokines (IL1β, IL6, and IL12a) and chemokines (CCL2, CCL3, CCL4, CCL5, and CXCL9) was significantly down‐regulated in the T1, T2, and T4 groups (Figure 4F). These combined results indicate that increased erythrocyte engulfment‐induced glycolysis suppression alleviated the inflammatory responses of macrophages.

The possible effects of enhanced phagocytosed erythrocytes on immune responses and bone regenerative events were finally investigated. The expression of core genes related to inflammatory responses was first examined (Figure S3D, Supporting Information) and their interactions were analyzed (Figure S3E, Supporting Information). The top 30 core genes in the T1, T2, and T4 groups were identified according to the degree to perform interaction analysis with bone development‐related genes respectively. Results showed that these top 30 core inflammatory genes in each group were closely correlated with bone development genes (Figure 4G). GO enrichment analysis further revealed that the correlated genes were enriched in the osteoblast differentiation term, ranking top 2 in significance for the T2 and T4 groups, and fourth for the T1 group (Figure 4G). These findings suggest that enhanced phagocytosed erythrocytes‐induced macrophage immune responses could influence bone regeneration, particularly in the T2 and T4 groups.

Collectively, enhanced erythrocyte engulfment‐induced glycolysis suppression inhibited M1 polarization, and reduced inflammatory responses, which could influence bone regeneration.

### 2.4 The Enhanced Bio‐Elimination of Erythrocytes Created Favorable Bone Regenerative Microenvironment In Vivo

As previously mentioned, longstanding compacted erythrocytes impair the infiltration and activity of MSCs.^[^ ^6^, ^7^, ^8^ ^]^ To assess whether “bio‐elimination of erythrocytes” could optimize the bone regenerative microenvironment in vivo, we first investigated the effect of DMOG on erythrocyte clearance and then characterized the local bone regenerative microenvironment, focusing on fibrin networks and inflammatory responses.

**Figure 5:**
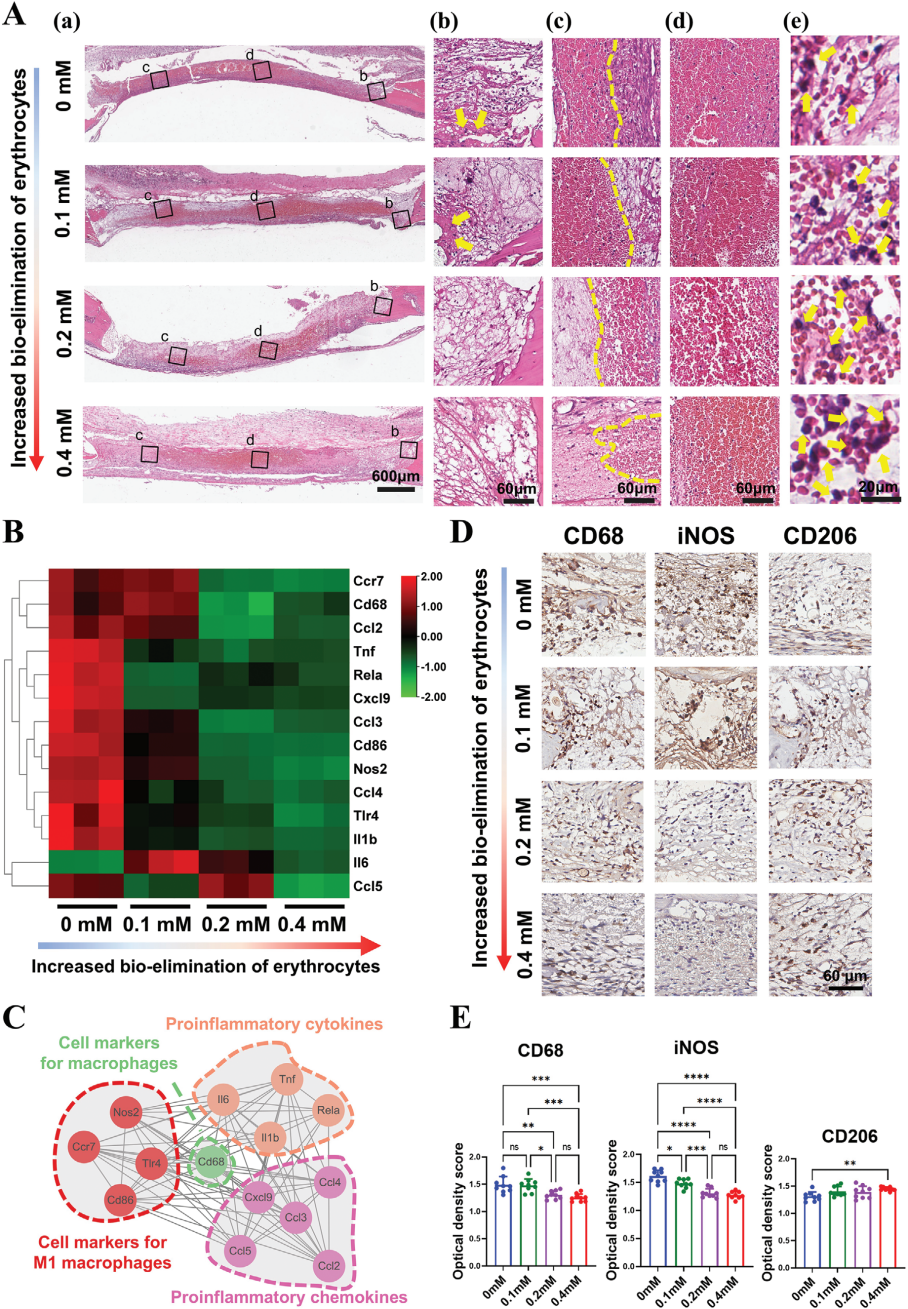
The effect of enhanced bio‐elimination of erythrocytes on the bone regenerative microenvironment in vivo. A) H&E images of bone defects in different groups 3 days after surgery. (a) Overview of the bone defect. The general deep red area where erythrocytes lay in DMOG‐treated groups was significantly smaller than that of the control group, indicating increased bio‐elimination of erythrocytes. (b) The magnification images at the edge of defects with yellow arrows indicating dense matted deposits. (c) The magnification images at the quarter site of defects with the dotted line indicating the boundary of the area occupied by erythrocytes. (d) The magnification images at the center of defects displayed compacted erythrocytes. (e) Representative images of macrophages that have phagocytosed erythrocytes (indicated by yellow arrows). B) Heatmap showed that gene expression of the typical inflammation‐related cytokines and chemokines within defects was significantly down‐regulated after increased bio‐elimination of erythrocytes (n = 3). C) Interaction analysis showed these inflammation‐related cytokines and chemokines were closely correlated with each other. D‐E) IHC staining and semi‐quantitative analysis results of the expression of the universal macrophage marker CD68, the M1 macrophage marker iNOS, and the M2 macrophage marker CD206 (n = 9). Data presented as mean ± SD; ^*^ *p *< 0.05, ^**^ *p *< 0.01, ^***^ *p *< 0.001, ^****^ *p *< 0.0001, and ns means not significant by one‐way ANOVA with Tukey's post hoc test.

Three days after surgery, erythrocyte clearance in blood clots was examined through hematoxylin and eosin (H&E) staining. In the control group, erythrocytes occupied the majority of the bone defect, indicating the dominant role of erythrocytes during bone healing (**Figure** **5**A‐a). Erythrocyte clearance began at the edge of the defect and progressed toward the center. The overall deep red area where erythrocytes were located in DMOG‐treated groups was significantly smaller compared to the control group (Figure 5A‐a; Figure S4, Supporting Information). Erythrocytes were nearly removed at the edge of defects in DMOG‐treated groups, while they were scattered in the control group (Figure 5A‐b). At the quarter site of defects, the area occupied by erythrocytes was significantly smaller in the 0.2 and 0.4 mm groups than in the 0 and 0.1 mm groups (Figure 5A‐c). At the center of defects, tightly packed erythrocytes were observed in all groups (Figure 5A‐d). Erythrophagocytosis of macrophages was more apparent in the 0.2 and 0.4 mm groups than in other groups (Figure 5A‐e). These results showed that in blood clots within rat calvarial defects, DMOG promoted macrophage‐mediated bio‐elimination of erythrocytes in a dose‐dependent manner.

In addition to erythrocyte clearance, alterations in fibrin networks were also observed. Three days after surgery, relatively thick fibrin and compacted fibrin meshes with dense matted deposits, attributed to erythrocyte interference,^[^ ^7^ ^]^ were observed at the edge of defects in the 0 and 0.1 mm groups. In contrast, thin fibrin and loose fibrin meshes without dense matted deposits were observed in the 0.2 and 0.4 mm groups (Figure 5A‐b). Similar results were noted at the quarter site of defects (Figure 5A‐c). However, the fibrin network was not visible at the center of defects due to the stacked erythrocytes that had not been phagocytosed (Figure 5A‐d). These results indicate that enhanced bio‐elimination of erythrocytes promotes the formation of a relatively loose fibrin network, which is beneficial for MSCs infiltration and bone regeneration.^[^ ^36^, ^37^ ^]^

Next, the effect of erythrocyte bio‐elimination on local inflammatory responses was determined. RT‐qPCR results showed a significant decrease in the gene expression of most inflammation‐related molecules in groups with increased erythrocyte bio‐elimination, particularly in the 0.2 and 0.4 mm groups (Figure 5B). These molecules, including pro‐inflammatory cytokines (IL6, IL1β, TNF‐α, P65) and chemokines (CCL2, CCL3, CCL4, CCL5, and CXCL9), and M1 polarization markers (CD68, CD86, TLR4, CCR7, iNOS), have a close interaction (Figure 5C). Immunohistochemistry (IHC) staining further showed that compared to the 0 mm group, the expression levels of CD68 and iNOS were significantly down‐regulated in the 0.1, 0.2, and 0.4 mm groups, while CD206 was slightly up‐regulated only in the 0.4 mm group (Figure 5D,E). These results indicate that enhanced erythrocyte bio‐elimination effectively limited macrophage M1 polarization and reduced local inflammatory responses.

In summary, erythrocytes occupied the majority of the bone defect area, creating an unfavorable bone regenerative microenvironment. DMOG‐induced hypoxia‐mimicking promoted erythrocyte clearance within blood clots in rat calvarial defects through macrophage erythrophagocytosis. Enhanced bio‐elimination of erythrocytes created a loose fibrin mesh without dense matted deposits and reduced local inflammation, thereby optimizing the bone regenerative microenvironment.

### 2.5 The Enhanced Bio‐Elimination of Erythrocytes Promoted Early Osteogenesis and Eventual Bone Regeneration

**Figure 6:**
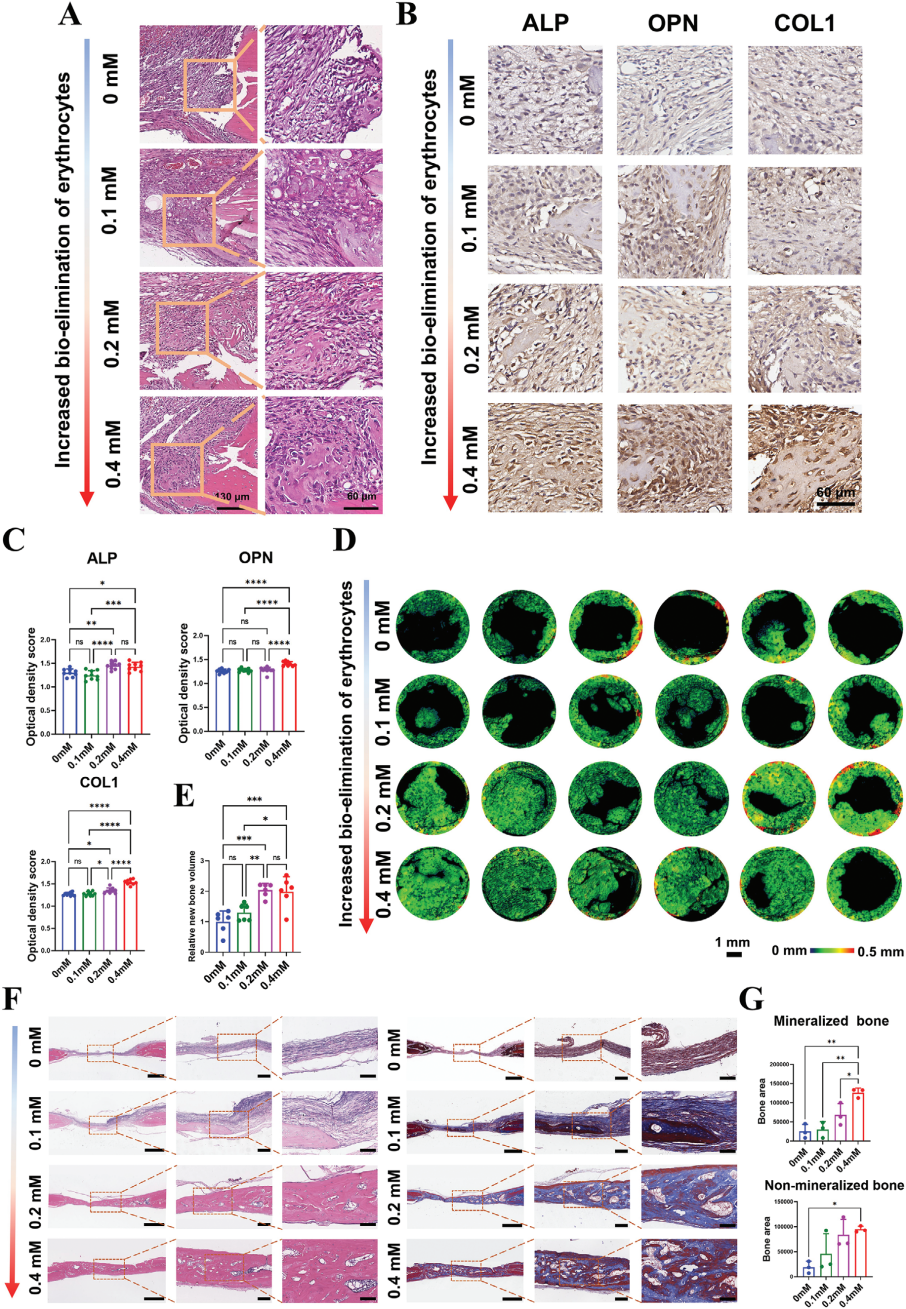
The effect of enhanced bio‐elimination of erythrocytes on the early osteogenesis and eventual bone regeneration. A) H&E images of edges of bone defects in different groups 5 days after surgery showed that DMOG‐mediated increased bio‐elimination of erythrocytes effectively facilitated osteogenesis. B‐C) IHC staining and semi‐quantitative analysis results of the expression of markers indicating osteoblast differentiation and osteogenesis, including ALP, OPN, and COL1 (n = 9). D) 3D reconstruction images of defects and E) Semi‐quantitative statistical analysis results of new bone volume showed apparent bone regeneration in 0.2 and 0.4 mm groups (n = 6). F) H&E staining (left, middle, and right scale bars = 1 mm, 200, and 100 µm, respectively) and Masson's trichrome staining (left, middle, and right scale bars = 1 mm; 200 and 100 µm, respectively) images of the selected centric sections of bone defects, and G) Semi‐quantitative analysis results of non‐mineralized bone and mineralized bone (n = 3). Data presented as mean ± SD; ^*^ *p *< 0.05, ^**^ *p *< 0.01, ^***^ *p *< 0.001, ^****^ *p *< 0.0001, and ns means not significant by one‐way ANOVA with Tukey's post hoc test.

Early osteogenesis was investigated 5 days post‐surgery via H&E and IHC staining. H&E images showed ongoing erythrocyte clearance, with some stacked erythrocytes accumulating in the middle of defects, and bone defects were filled with randomly arranged soft tissues (Figure S5, Supporting Information). However, different events were observed at the edge of defects where erythrocyte clearance began first. In the 0 and 0.1 mm groups, fibrous‐like tissue with thin collagen fibers and small, long spindle‐shaped cells were observed (**Figure** **6**A). IHC images further revealed that there were few ALP, OPN, or COL1 positive cells, especially in the 0 mm group (Figure 6B,C). These results indicated that bone regeneration was significantly hindered due to insufficient erythrocyte clearance. In contrast, nascent bone matrix and thick collagen fibers were observed at the edge of defects in the 0.2 and 0.4 mm groups, embedded with various cells in spindle, oval, or oblong shapes (Figure 6A). IHC staining showed many of these cells were ALP, OPN, or COL1 positive (Figure 6B,C). Taken together, these results showed that compared to the 0 and 0.1 mm groups where erythrocyte clearance was inadequate, there were enhanced MSCs infiltration, osteoblast differentiation, and collagen secretion in the 0.2 and 0.4 mm groups, indicating that enhanced bio‐elimination of erythrocytes significantly promoted early osteogenesis.

Four weeks post‐surgery, Micro‐CT 3D reconstruction images revealed minimal bone generation within calvarial defects in the 0 and 0.1 mm groups, with the defect centers nearly empty. In contrast, apparent new bone formation was observed in the 0.2 and 0.4 mm groups (Figure 6D), which was further supported by the semi‐quantitative statistical analysis of new bone volume (Figure 6E). H&E images revealed that bone defects in the 0 mm group were dominated by fibrous tissue, with only a few new bones present at the defect edge. Although bone‐like tissues slightly increased in the 0.1 mm group, the majority of the defect region was still occupied by fibrous tissue. In contrast, bone defects in the 0.2 and 0.4 mm groups were filled with confluent bone with larger new bone (Figure 6F), which aligned with the Micro‐CT results. Masson's trichrome staining results further showed significantly more mineralized bone in the 0.2 and 0.4 mm groups compared to the other groups (Figure 6F,G), indicating enhanced bone maturation.

Taken together, these results showed that an optimized bone regenerative microenvironment created by enhanced bio‐elimination of erythrocytes effectively promoted early osteogenesis, late new bone regeneration and maturation.

### 2.6 Implications for Bone Regeneration‐Regulating Strategies and Advanced Biomaterial Development Focusing on the Bio‐Elimination of Erythrocytes

**Figure 7:**
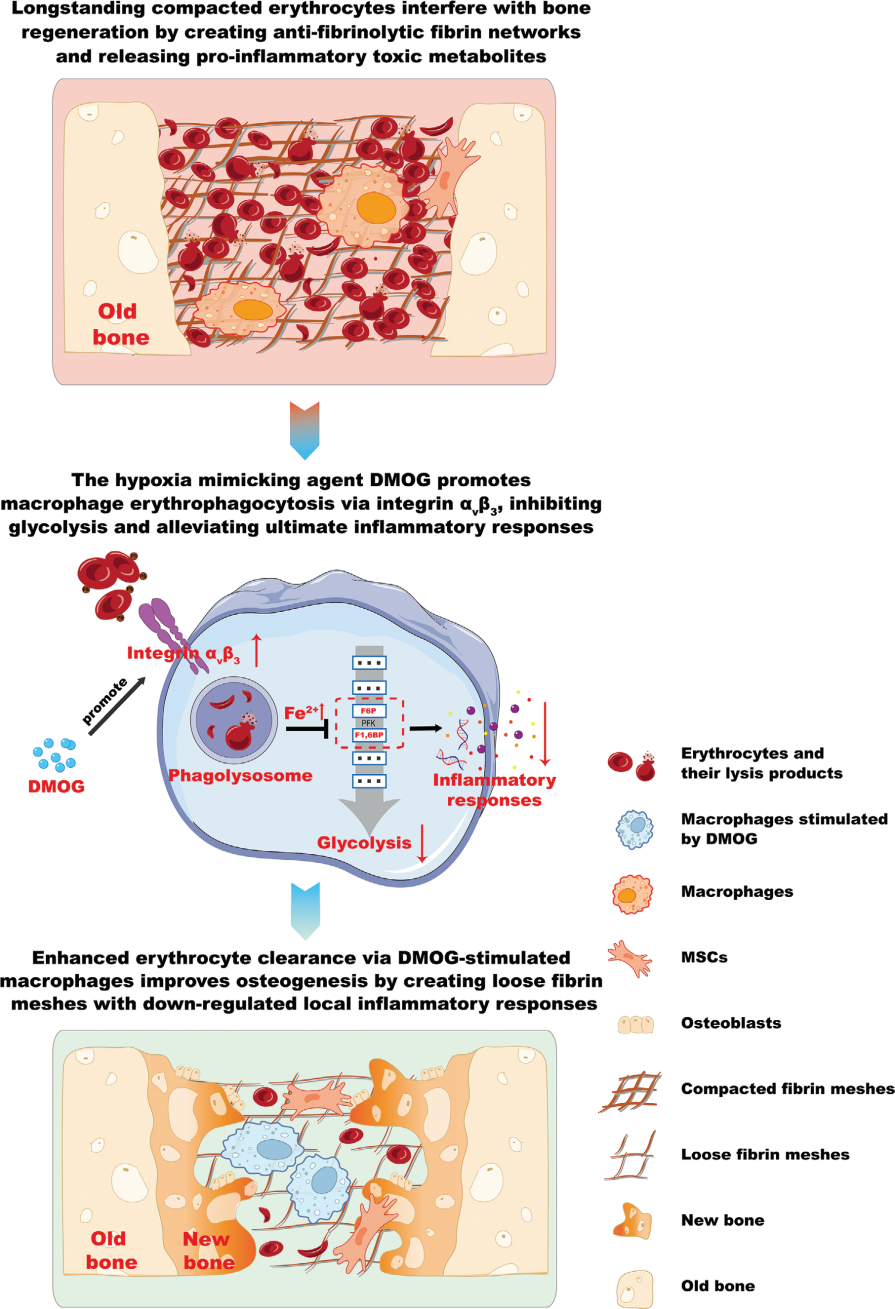
Schematic figure shows the effects of hypoxia‐mimicking on macrophage‐elimination of erythrocytes, and the subsequent immunomodulation and osteogenesis. Macrophage‐elimination of erythrocytes was enhanced by DMOG regulating integrin α_v_β_3_. The phagocytosed erythrocytes modulated the intracellular Fe^2+^‐glycolysis interaction, thus reducing macrophage inflammatory responses. Enhanced bio‐elimination of erythrocytes in blood clots created an optimized bone regenerative microenvironment characterized by loose fibrin mesh without dense matted deposits and reduced local inflammation, which promoted bone regeneration. PS: phosphatidylserine. Schematics were created with BioRender.com.

As aforementioned, erythrocytes are the dominant cells in blood clots and can affect the bone regeneration process, which has been ignored by researchers for a long time. Bio‐elimination of erythrocytes should be a promising strategy for improving bone regeneration outcomes. Although precise regulation of erythrocyte clearance is difficult, we succeeded in promoting erythrocyte removal through hypoxia‐mimicking‐induced enhanced erythrophagocytosis by macrophages. Phagocytosed erythrocytes then suppressed the glycolysis of macrophages by tuning Fe^2+^‐phosphofructokinase, creating a down‐regulated inflammatory microenvironment with loose fibrin mesh in blood clots, and as a result, bone regeneration was enhanced (**Figure** **7**). This highlighted the significance of bio‐elimination of erythrocytes during bone regeneration and supported the feasibility of modulating macrophage‐elimination of erythrocytes to overcome bone regeneration hindrance attributed to erythrocytes, thus facilitating new bone formation.

In this study, it was shown that the hypoxia‐mimicking agent DMOG promoted erythrophagocytosis of macrophages primarily by up‐regulating integrin α_v_β_3_. DMOG also influenced other erythrophagocytosis‐related receptors, including AXL, MERTK, and TIMD4. The underlying mechanism by which DMOG regulates the expression of phagocytosis‐related receptors probably involves activation of HIF‐1α.^[^ ^12^, ^13^, ^38^, ^39^ ^]^ Other hypoxia‐mimicking agents may similarly regulate these receptors and promote macrophage erythrophagocytosis, though their effects could vary due to differences in ways of HIF‐1α activation, safe concentration thresholds, and specificities. The elevated intracellular ferrous iron ion could trigger complex intracellular reactions. In addition to the primary down‐regulation of glycolysis and M1 polarization, other intracellular reactions and the consequent function changes of macrophages such as M2 polarization could also occur, which warrant further investigation. In vivo, the observed outcomes are a combination of multiple effects. Its promotion of osteogenic differentiation in mesenchymal stem cells^[^ ^40^ ^]^ may contribute to these outcomes. Additionally, DMOG‐stimulated macrophages not only phagocytize erythrocytes but may also engulf apoptotic neutrophils,^[^ ^41^ ^]^ which could also contribute to improved bone regeneration. However, the specific effects require further study.

**Figure 8:**
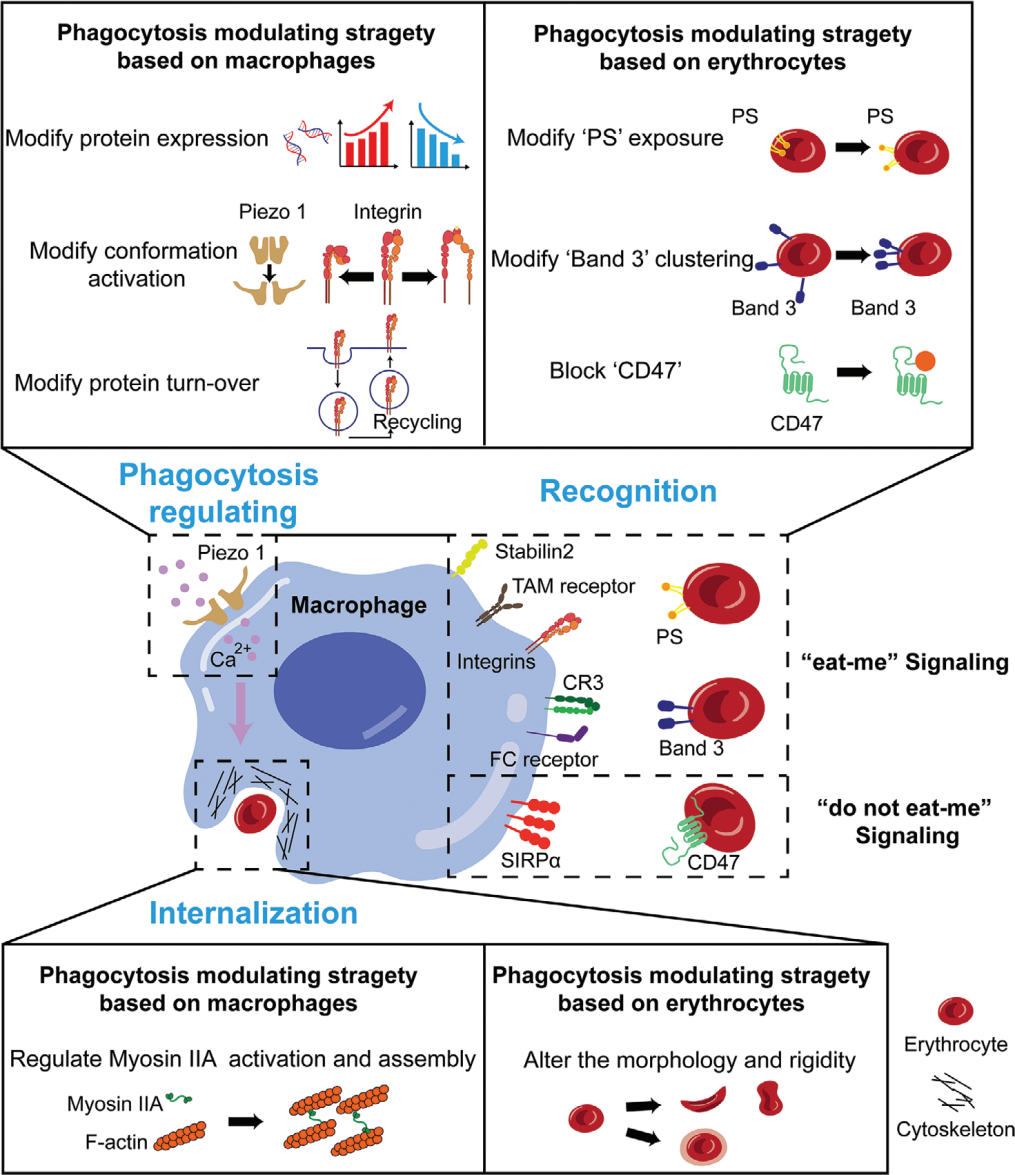
Schematic diagram displays the strategy for manipulating erythrophagocytosis of macrophages to guide the development of advanced biomaterials focusing on the bio‐elimination of erythrocytes. Erythrocyte phagocytosis includes the recognition between phagocytosis‐related receptors on the macrophage membrane and ligands on erythrocyte surface and the subsequent internalization, as well as the regulation‐mediated by Piezo1‐mediated Ca^2+^ influx. Stabilin2, TAM receptors (AXL, MERTK), and integrins (integrin α_V_β_3_, integrin α_3_β_1_) can bind the externalized phosphatidylserine, and complement receptor 3 and Fc receptor are able to bind clustered band 3 to initiate the “eat‐me” signaling, while SIRPα binding CD47 can trigger “do not eat‐me” signaling. The internalization process involves not only the activity of myosin IIA of macrophages but also the shape and stiffness of erythrocytes. Based on macrophages, developing biomaterials that modify the expression, conformation, or turn‐over of phagocytosis‐promoting, phagocytosis‐inhibiting, and phagocytosis‐regulating proteins or the activation and assembly of myosin IIA can regulate the macrophage‐elimination of erythrocytes. Based on erythrocytes, developing biomaterials that modify the phosphatidylserine exposure and the band 3 clustering, block the CD47, or alter the morphology and rigidity of erythrocytes can also modulate the macrophage‐elimination of erythrocytes. PS: phosphatidylserine; TAM: Tyro3, Axl, and Mertk; CR3: complement receptor 3; SIRPα: signal regulatory protein alpha; Schematics were created with BioRender.com.

Erythrocyte phagocytosis involves the recognition between phagocytosis‐related receptors on the macrophage membrane and ligands on the erythrocyte surface, subsequent internalization, and regulation‐mediated by Piezo1‐mediated Ca^2+^ influx.^[^ ^42^ ^]^ To manipulate erythrocyte phagocytosis, potential modulating strategies based on macrophages and erythrocytes have been proposed and summarized in **Figure** **8**, which could guide the development of advanced biomaterials for erythrocyte clearance. By targeting key molecules in phagocytosis recognition, internalization, or regulation, advanced biomaterials that manipulate erythrocyte phagocytosis can be developed. For example, based on macrophages, biomaterials that modify the expression, conformation, or turnover of phagocytosis‐related membrane proteins can modulate the balance between “eat‐me” signaling and “do‐not‐eat‐me” signaling, thus manipulating phagocytosis. Based on erythrocytes, biomaterials that alter the morphology or rigidity of erythrocytes can tune the internalization process,^[^ ^43^ ^]^ thus modulating phagocytosis.

Although local hypoxia develops following a bone injury, it is often insufficient to induce adequate erythrocyte clearance in time, resulting in impaired bone regeneration. The application of the hypoxia‐mimicking agent DMOG successfully accelerated macrophage‐elimination of erythrocytes, ultimately improving bone regeneration. These indicate that hypoxia‐mimicking biomaterials can be a future direction for developing advanced materials focusing on erythrocyte clearance to improve bone regeneration. This study discovered a new biological role of hypoxia‐mimicking in regulating macrophage‐mediated erythrocyte clearance, offering deeper insights into the hypoxia‐mimicking‐mediated bone regeneration, which aligns with the “blood‐activating and stasis‐transforming” concept in Traditional Chinese Medicine.^[^ ^44^ ^]^ Specifically, hypoxia‐mimicking treatment could hasten the elimination of compacted erythrocytes that form a physical barrier, thus promoting the entry of new blood and cells essential for tissue regeneration, resulting in an activated, favorable microenvironment instead of a stagnant one. Moreover, release‐controlling and sustained‐release hypoxia‐mimicking biomaterials can be further developed to precisely regulate erythrocyte phagocytosis by manipulating the hypoxia degree spatiotemporally.

## 3 Conclusion

The hypoxia‐mimicking agent DMOG enhanced macrophage elimination of erythrocytes mainly by up‐regulating the expression of integrin α_v_β_3_. The phagocytosed erythrocytes decreased macrophage inflammatory responses by regulating intracellular Fe^2+^‐phosphofructokinase‐glycolysis. Bio‐elimination of erythrocytes in vivo made a loose fibrin mesh without dense matted deposits and reduced local inflammation, thus creating an optimized bone regenerative microenvironment that effectively promoted bone regeneration. Modulating macrophage elimination of erythrocytes could be a promising and valuable strategy for overcoming erythrocyte‐caused bone regeneration hindrance.

## 4 Experimental Section

### Cell Culture

RAW 264.7 cells (referred to as RAW cells hereafter) were selected as the model cell to research macrophage erythrophagocytosis. RAW cells were cultured in a complete medium composed of Dulbecco's Modified Eagle's Medium (DMEM, Gibco, USA), 10% fetal bovine serum (Gibco, USA), and 1% penicillin/streptomycin (Gibco, USA), and culture flasks or plates were placed in cell incubators (37 °C, 5% CO2).

### Cytotoxicity Assay of DMOG

Cell‐Counting Kit‐8 (CCK8) (#CK04, Dojindo, Japan) was used to assess the cytotoxicity of DMOG on RAW cells. After the stimulation, DMOG solutions were removed and replaced with a culture medium containing 10% CCK8 reagent, followed by incubation at 37 °C for another 1 h. Then the solution in each well was transferred to another new plate and its absorbance at 450 nm was measured using a microplate reader. DMOG was further loaded into blood clots that were implanted into rat calvarial bone defects to assess the in vivo systemic toxicity of DMOG.

### Erythrocytes Isolation, Fluorescence Labeling, and Eryptosis Induction

Erythrocytes were isolated from the blood of BALB/c mice. All experimental procedures were reviewed and approved by the Institutional Animal Care and Use Committee of Sun Yat‐Sen University and performed following the principles of animal protection, animal welfare, and ethics (approval NO. SYSU‐IACUC‐2023‐001132). Eight‐to‐nine‐week‐old BALB/c mice were anesthetized by intraperitoneal injection with 2% pentobarbital sodium at a ratio of 2.5 mL kg^−1^. The whole blood was collected as previously described.^[^ ^45^ ^]^ Then the whole blood was washed with ice‐cold PBS followed by centrifugation at 500 rcf until the supernatant was clear. The red erythrocyte deposits were collected for subsequent experiments. Erythrocytes were labeled with a Cell Plasma Membrane Staining Kit with Dil (Beyotime, China) according to the manufacturer's instructions. Eryptosis was induced by 56 °C heat shock for 5 min in a water bath according to a previous report.^[^ ^46^ ^]^

### Evaluation of the Influence of DMOG on Erythrophagocytosis In Vitro

Within the safe concentrations, RAW cells were pre‐stimulated with DMOG for 24 h and then subjected to RT‐qPCR to detect the expression of phagocytosis‐related genes. Primers applied in this part are listed in Table S1 (Supporting Information). Up‐regulated genes were submitted to DAVID Bioinformatics Resources, and GO enrichment was performed to analyze their main functions. Data visualization was performed using SangerBox 3.0 (http://vip.sangerbox.com/home.html). The interaction of these upregulated molecules was performed through the String database. RAW cells pre‐stimulated with DMOG for 24 h were co‐cultured with heat‐shocked erythrocytes in a new complete medium. High content cell real‐time analysis system (PerkinElmer, USA) and flow cytometric analysis were used to detect macrophage erythrophagocytosis at 2, 8, and 16 h after co‐culture, respectively.

### Investigation of Effects of Enhanced Erythrophagocytosis on Macrophages

After co‐culture with erythrocytes for 16 h, RAW cells were divided into T0, T1, T2, and T4 groups respectively, with each group having three biological duplicates. These RAW cells were subjected to RNA‐seq, RT‐qPCR, and Immunofluorescence staining. Primers applied in this part are listed in Table S1 (Supporting Information).

### RNA‐Seq

Total RNA of RAW cells was extracted. Quality evaluation was performed through a fragment analyzer. According to the 150‐bp paired‐end run protocol provided by BGI genomics (Shenzhen, China), the cDNA library was constructed and sequenced on a BGISEQ‐500 platform. Low‐quality reads were excluded, and clean reads were mapped to the mouse reference genome using Bowtie2 and HISAT. Gene expression levels were expressed as fragments per kilobase of exon model per million mapped fragments (FPKM).

### Bioinformatic Analysis

Principal Component Analysis was applied to assess the general differences among the four groups. Differential expression analysis was carried out through the R package DESeq2. Differentially expressed genes between two different groups were used for KEGG pathway enrichment analysis through DAVID Bioinformatics Resources. Protein‐protein interactions (PPI) were obtained from the STRING database. The interaction network was loaded into Cytoscape, and the core sub‐network was sorted out using MCODE plug‐in, with the degree calculated. GSEA was performed to analyze the differences in target gene sets between two groups.^[^ ^47^ ^]^ Target genes were subjected to DAVID Bioinformatics Resources and GO enrichment was performed. Venn diagrams were used to identify intersected genes among several target terms. Gene set variance analysis (GSVA) scores of target gene sets for each sample were calculated using R package GSVA, and were used for Spearman's rank correlation analysis to assess the correlation of two target gene sets. All data visualization was performed with OmicStudio tools at https://www.omicstudio.cn/tool, GraphPad Prism, TBtools, and Cytoscape.

### Immunofluorescence Staining

RAW cells that had engulfed erythrocytes were fixed with 4% paraformaldehyde (Beyotime, China) and then washed with PBS. These RAW cells were blocked with 1% bovine serum albumin (BSA; Amresco, USA) in PBS and then incubated with CCR7 (#ab32527, Abcam, UK) antibody at 4 °C overnight. After washing with PBS, RAW cells incubated with Alexa Fluor 488 (#EM35141‐01, EMAR, China) fluorescent secondary antibody at room temperature for 1 h, followed by PBS wash. RAW cells were then stained with 4,6‐diamino‐2‐phenyl‐indole (DAPI; Beyotime, China) for 10 min. The fluorescent images were obtained through a confocal laser scanning microscope (Olympus FV3000, Japan). The intracellular content of Fe^2+^ was detected by a FerroOrange staining kit (#F374, Dojindo, Japan) according to the manufacturer's instructions. Semi‐quantitative analyses were performed with Image‐J.

### Animal Surgery

Eight to nine weeks old SD rats were subjected to in vivo experiments. All the surgical procedures were reviewed and approved by the Institutional Animal Care and Use Committee, Sun Yat‐Sen University, and met the requirements of animal protection, animal welfare and ethics (approval NO. SYSU‐IACUC‐ 2022‐000153). The rats were anesthetized by intraperitoneal injection with 2% pentobarbital sodium at a ratio of 2.5 ml kg^−1^. Following the previously reported typical surgical procedures,^[^ ^48^ ^]^ two circle defects of 5 mm diameter were separately created on the left and right side of the rat calvaria with a drill. Blood clots with DMOG at different concentrations were prepared according to our previous study.^[^ ^49^ ^]^ Briefly, blood was collected from the tail vein and mixed with DMOG normal saline solutions (0, 1, 2, 4 mm) in a 9:1 volume ratio to obtain clots with DMOG at final concentrations of 0, 0.1, 0.2, and 0.4 mm. Each clot was made into 200 µL in a round container. After coagulation, these clots were separately implanted into circle calvarial bone defects and a layered suturing was performed to close the wound. The calvaria were collected as samples for histological analysis at different time points.

### Erythrophagocytosis Assay In Vivo

To evaluate the influence of DMOG on erythrophagocytosis in vivo, rats were sacrificed with excessive anesthesia 3 days after implantation. Calvaria samples were fixed with 4% paraformaldehyde. After decalcification, these samples were embedded in paraffin and 4 µm thick sections were generated using a Leica Biosystems RM2255 (Leica, Germany) for subsequent histological assay. For H&E staining, briefly, after dewaxing and hydration, Mayer's hematoxylin was applied to stain cell nuclei and eosin was applied to stain cell plasm and extracellular matrix on selected slides.

### Characterization of The Local Inflammatory Microenvironment In Vivo

Three days after the surgery, macrophage polarization within and near the defects was first investigated through immunohistochemical (IHC) staining of CD68, iNOS, and CD206. After dewaxing and hydration, the selected sections were incubated with 3% H_2_O_2_ for 30 min to eliminate endogenous peroxidase activity, blocked with 1% BSA at room temperature for 1 h, and then incubated with CD68 (#ab125212, Abcam, UK), iNOS (#ab283655, Abcam, UK), and CD206 (#ab64693, Abcam, UK) antibodies at 4 °C overnight. After that, the slides were incubated with a goat anti‐rabbit secondary antibody (Gene Tech, China) at room temperature for 1 h. The antibody‐antigen complex was visualized by adding diaminobenzidine solution (Gene Tech, China). The slides were then counterstained with Mayer's hematoxylin for 2 min, dehydrated, and mounted. Semi‐quantitative statistics of positive cells in images were performed using Image‐J with an IHC profiler plug‐in. To detect the gene expression profile of typical inflammatory‐related cytokines or chemokines at the defects, blood clots within calvarial defects were collected 3 days after the surgery and total RNA of the clot tissue was extracted using TRIZOL (Beyotime, China) and the expression of inflammation‐related molecules was detected through RT‐qPCR. Primers applied in this part are listed in Table S1 (Supporting Information).

### Evaluation of Bone Regeneration

Five days after the surgery, H&E staining was applied to observe osteogenesis, and IHC staining of alkaline phosphatase (ALP) (#ab65834, Abcam, UK), osteopontin (OPN) (#bs‐0026R, Bioss, China), and collagen I (COL I) (#ab34710, Abcam, UK) was applied to further verify cell types embedded in the new bone matrix. Four weeks after the surgery, calvaria were scanned through a Micro‐CT scanner (µCT50; SCANCO Medical AG, Switzerland) at a resolution of 10 µm, a source voltage of 70 kV, and a current of 114 µA. 3D reconstruction images of the bone defects were generated. The new bone volume was calculated using the software Materialise Mimics Research 19.0 (Materialise, Belgium). H&E staining was further used to observe the new bone formation and new bone mineralization. Masson's trichrome staining was performed with a Masson's trichrome staining kit (Solarbio, China) according to the manufacturer's instructions.

### Statistical Analysis

Each experiment was repeated at least three times, and the sample size (n) was noted in the figures. Experimental results were presented as mean ± standard deviations. For statistical analysis, GraphPad Prism was used and the built‐in One‐way ANOVA was performed followed by recommended Tukey's multiple comparison post hoc test in all cases. A *P* value of less than 0.05 was considered significant (without further explanation, ^*^ *p *< 0.05, ^**^ *p *< 0.01, ^***^ *p *< 0.001, ^****^ *p *< 0.0001, ns means not significant).

## Conflict of Interest

The authors declare no conflict of interest.

## Supporting information



Supporting Information

## Data Availability

Research data are not shared.
