# Supplementary material for: Hypoxia‐Mimicking Mediated Macrophage‐Elimination of Erythrocytes Promotes Bone Regeneration via Regulating Integrin αvβ3/Fe2+‐Glycolysis‐Inflammation
Source: Adv Sci (Weinh). 2024 Oct 1;11(45):2403921. doi: 10.1002/advs.202403921 (PMC11615788; doi:10.1002/advs.202403921)
Supplement: Supplementary file 1 — Supporting Information [file ADVS-11-2403921-s001.docx]

**Hypoxia Mimicking Mediated** **Macrophage-Elimination of Erythrocytes Promotes Bone Regeneration via Regulating Integrinα_v_β_3_ /Fe^2+^-Glycolysis-Inflammation**

*Yong Ao*^#^, *Yuanlong Guo*^#^, *Yingye Zhang*^#^, *Lv Xie*, *Ruidi Xia*, *Jieyun Xu*, *Mengru Shi*, *Xiaomeng Gao*, *Xiaoran Yu*, *Zetao Chen*^*^

**^#^**These authors contributed equally to this manuscript.

*Corresponding author: Professor Zetao Chen

Y. Ao, Y. Guo, Y. Zhang, L. Xie, R. Xia, J. Xu, M. Shi, X. Gao, X. Yu, Z. Chen

Hospital of Stomatology, Guanghua School of Stomatology, Sun Yat-sen University, Guangdong Research Center for Dental and Cranial Rehabilitation and Material Engineering, Guangzhou, 510055, China.

Email (Zetao Chen): chenzet3@mail.sysu.edu.cn


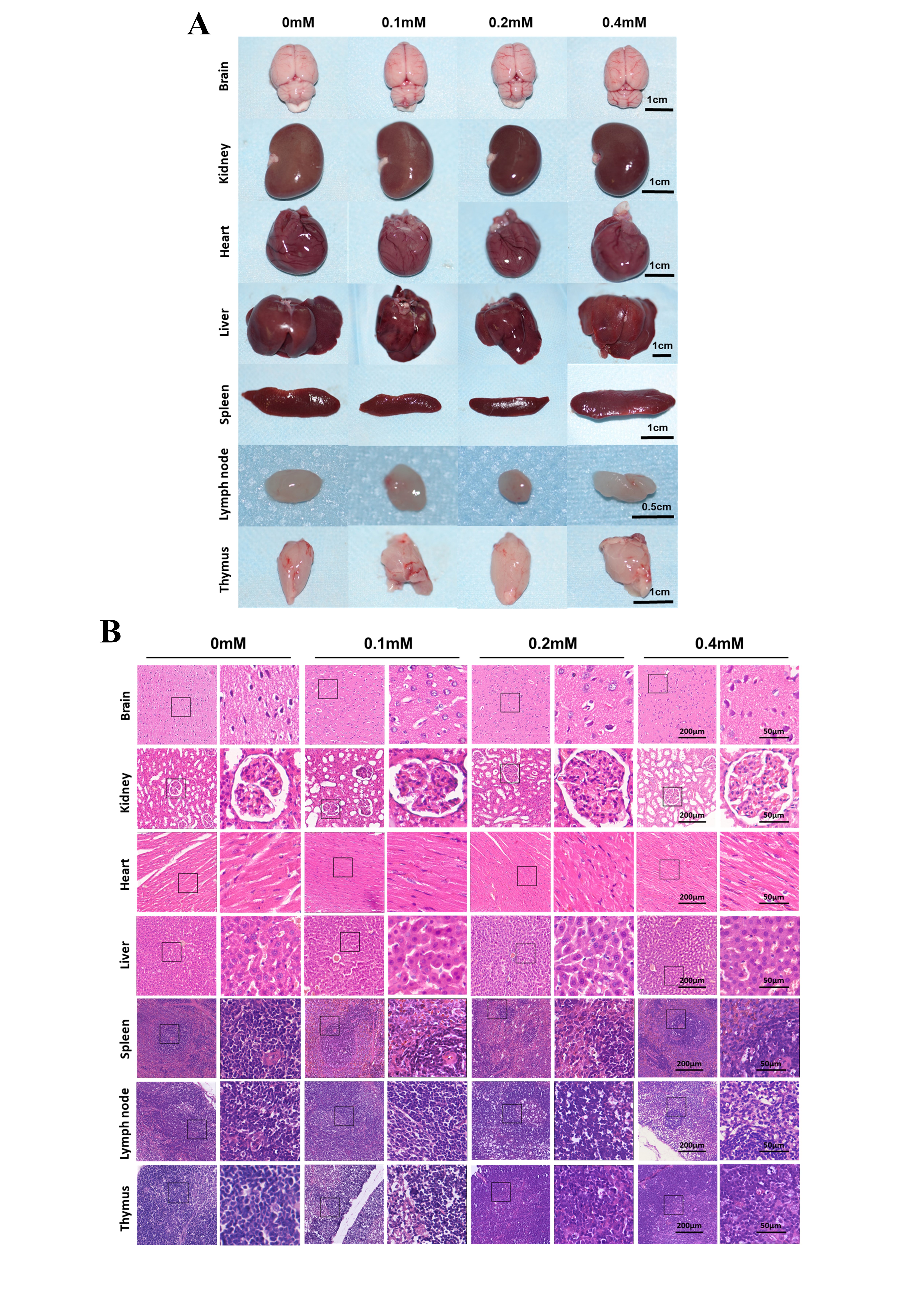


**Supplementary Figure 1.** Evaluation of systemic toxicity of DMOG in vivo. A) Macroscopic observation of the organs including the brain, kidney, heart, liver, spleen, lymph node, and thymus. B) Microscopic observation of organs, including the brain, kidney, heart, liver, spleen, lymph node, and thymus, through H&E staining.


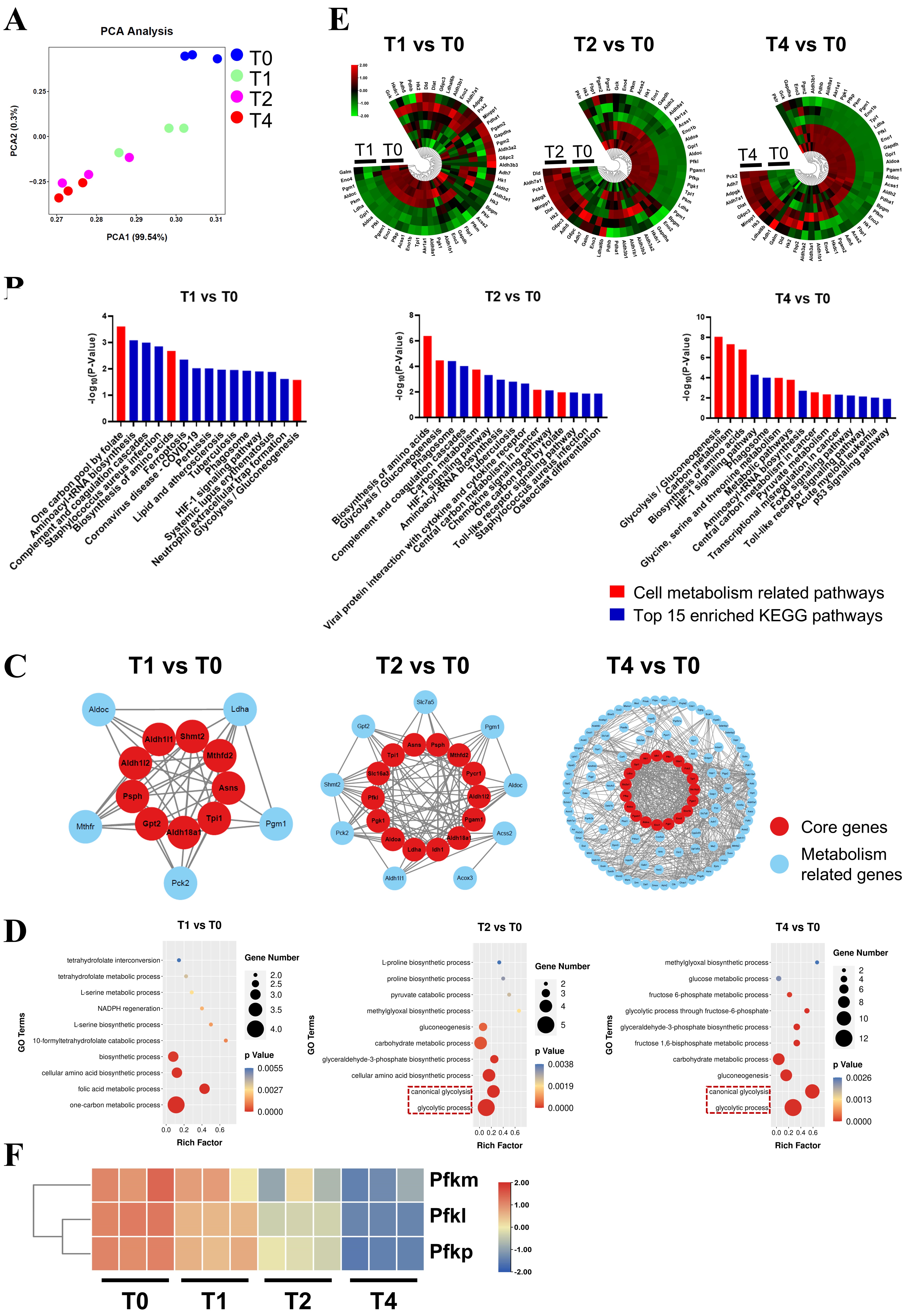


**Supplementary Figure 2.** Alternation of intracellular reactions of macrophages after increased erythrocyte engulfment. A) Principal component analysis of the transcriptomic results. B) KEGG enrichment results of differential genes in T1 vs. T0, T2 vs. T0, and T4 vs. T0, respectively. C) Interaction of cell metabolism-related genes. D) GO enrichment analysis results of the core genes of cell metabolism-related genes. E) Expression heatmap of genes in the glycolysis pathway in KEGG. F) Gene expression heatmap of *Pfkm*, *Pfkl*, and *Pfkp*.


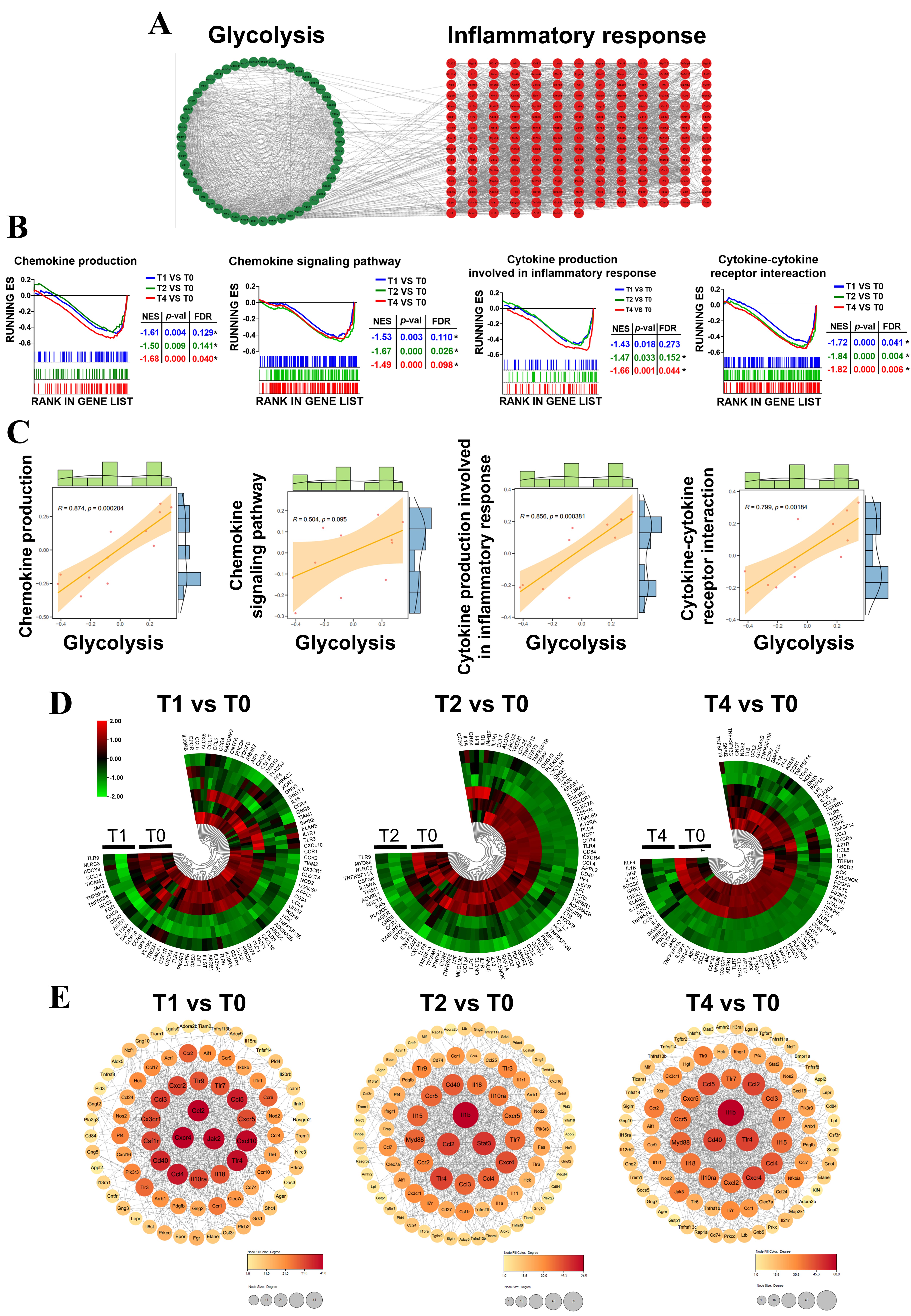


**Supplementary Figure 3.** Down-regulation of macrophage inflammatory responses after increased erythrocyte engulfment. A) Interaction analysis of glycolysis-related genes and inflammatory response-related genes. B) GSEA enrichment results of the macrophage inflammatory response-related gene sets, including “chemokine production”, “chemokine signaling pathway”, “cytokine production involved in inflammatory response”, and “cytokine-cytokine receptor interaction” (*|NES| > 1, P < 0.05, and FDR < 0.25). C) Spearman’s rank correlation analysis results of glycolysis and the above four macrophage inflammatory response-related gene sets. D) Expression heatmap of leading genes of the macrophage inflammatory response-related terms in GSEA analysis. E) Interaction analysis of leading genes of the inflammatory response-related terms in GSEA analysis.


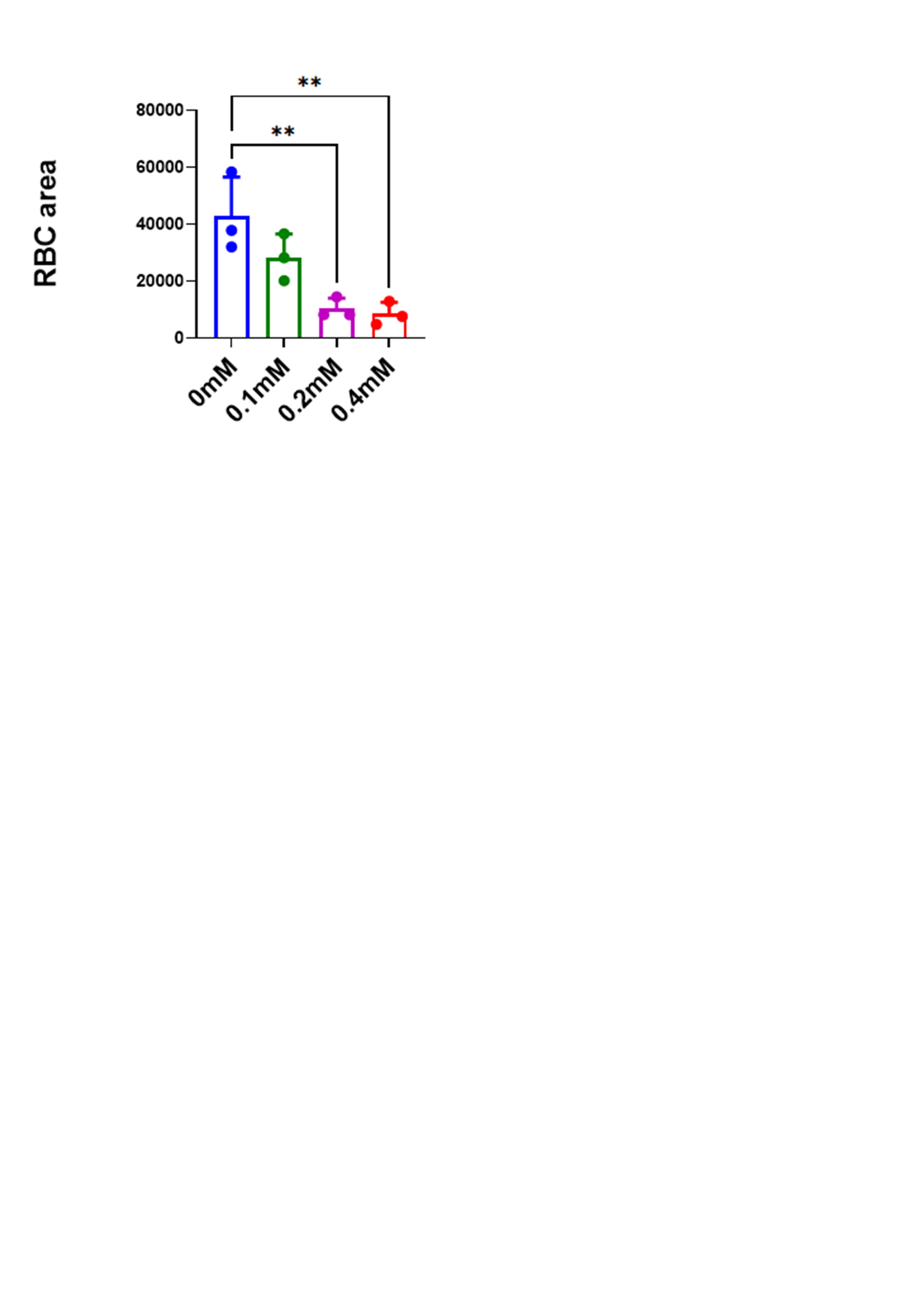


**Supplementary Figure 4.** The semi-quantitative statistical analysis results of the area occupied by erythrocytes in the 0 mm, 0.1 mm, 0.2 mm, and 0.4 mm groups. Data presented as mean ± SD; **p < 0.01 by one-way ANOVA with Tukey’s post hoc test.


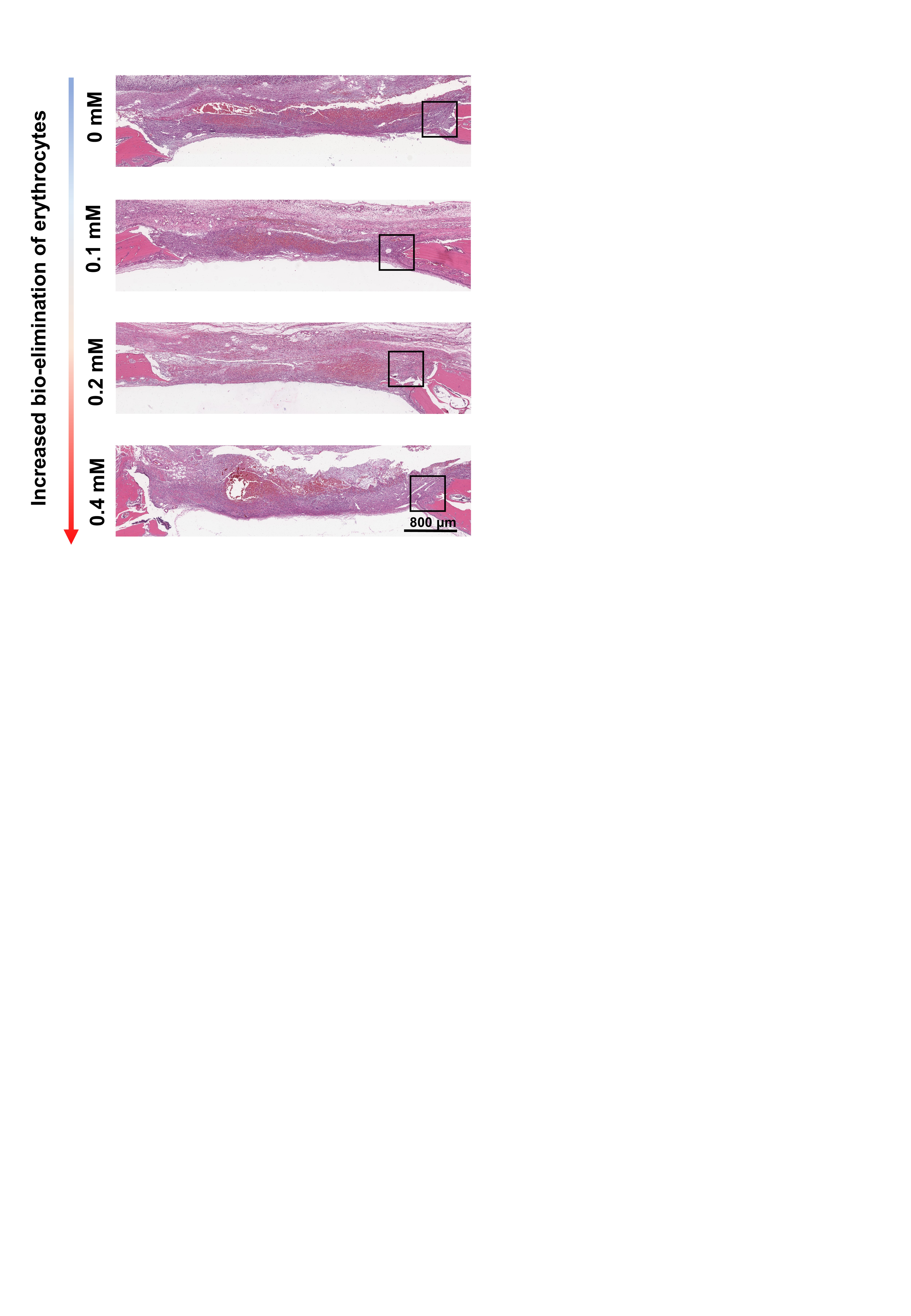


**Supplementary Figure 5.** General view of the H&E images of the selected centric sections of bone defects 5 days after surgery.

**Supplementary Table1. RT-qPCR primers applied in this study.**

| **Genes** | **Primer sequences (5’-3’)** |
| --- | --- |
| g-Actb | Forward: GACTGTTACTGAGCTGCGTTT |
|  | Reverse: AGGGTGAGGGACTTCCTGTA |
| m-Axl | Forward: GGAACCCAGGGAATATCACAGG |
|  | Reverse: AGTTCTAGGATCTGTCCATCTCG |
| m-Itgav | Forward: TTGATTCAACAGGCAATCGAGA |
|  | Reverse: AGCATACTCAACGGTCTTTGTG |
| m-Mfge8 | Forward: AGATGCGGGTATCAGGTGTGA |
|  | Reverse: GGGGCTCAGAACATCCGTG |
| m-Itgb5 | Forward: CTTACCCTGGTCAGAGGAAGTG |
|  | Reverse: CCTCAAGGTGAAAGACTGTGCTG |
| m-Itgb3 | Forward: CCCCGATGTAACCTGAAGGAG |
|  | Reverse: GAAGGGCAATCCTCTGAGGG |
| m-Mertk | Forward: TGCGTTTAATCACACCATTGGA |
|  | Reverse: TGCCCCGAGCAATTCCTTTC |
| m-Gas6 | Forward: CCGCGCCTACCAAGTCTTC |
|  | Reverse: CGGGGTCGTTCTCGAACAC |
| m-Thbs1 | Forward: GGTAGCTGGAAATGTGGTGCGT |
|  | Reverse: GCACCGATGTTCTCCGTTGTGA |
| m-Timd4 | Forward: AGCTTCTCCGTACAGATGGAA |
|  | Reverse: CCCACTGTCACCTCGATTGG |
| m-Cd300lb | Forward: GGTATCCCGCTGAGATTTGGA |
|  | Reverse: TCTTGTTGGTTTGCCATCTTGA |
| m-Cd36 | Forward: ATGGGCTGTGATCGGAACTG |
|  | Reverse: TTTGCCACGTCATCTGGGTTT |
| m-Il1b | Forward: TGGAGAGTGTGGATCCCAAG |
|  | Reverse: GGTGCTGATGTACCAGTTGG |
| m-Cd86 | Forward: CTGGACTCTACGACTTCACAATG |
|  | Reverse: AGTTGGCGATCACTGACAGTT |
| m-Tlr4 | Forward: GCCTTTCAGGGAATTAAGCTCC |
|  | Reverse: GATCAACCGATGGACGTGTAAA |
| m-Il6 | Forward: CTGCAAGAGACTTCCATCCAG |
|  | Reverse: AGTGGTATAGACAGGTCTGTTGG |
| m-Il12a | Forward: CATCCAGCTGCTCCTCTCAG |
|  | Reverse: GGCCGAAGTGAGGTGGTTTA |
| m-Ccl2 | Forward: AGGTGTCCCAAAGAAGCTGT |
|  | Reverse: AAGACCTTAGGGCAGATGCAG |
| m-Ccl3 | Forward: CAGCGAGTACCAGTCCCTTT |
|  | Reverse: GCAGTGGTGGAGACCTTCAT |
| m-Ccl4 | Forward: CCCAGCTCTGTGCAAACCTA |
|  | Reverse: GAGCAAGGACGCTTCTCAGT |
| m-Ccl5 | Forward: CGCACCTGCCTCACCATATG |
|  | Reverse: CTTCGAGTGACAAACACGACTGC |
| m-Cxcl9 | Forward: CATAATCTCAGACGGCAAAT |
|  | Reverse: CCACACTTCCACTCGCAGAAT |
| m-Hk1 | Forward: CAAGAAATTACCCGTGGGATTCA |
|  | Reverse: CAATGTTAGCGTCATAGTCCCC |
| m-Aldoa | Forward: AGTCCACCGGAAGCATTGC |
|  | Reverse: CAGCCCCTGGGTAGTTGTC |
| m-Pgk1 | Forward: GATGCTTTCCGAGCCTCACTGT |
|  | Reverse: ACCAGCCTTCTGTGGCAGATTC |
| m-Eno1 | Forward: TACCGCCACATTGCTGACTTGG |
|  | Reverse: GCTTGTTGCCAGCATGAGAACC |
| m-Pkm | Forward: CAGAGAAGGTCTTCCTGGCTCA |
|  | Reverse: GCCACATCACTGCCTTCAGCAC |
| m-Ldha | Forward: ACGCAGACAAGGAGCAGTGGAA |
|  | Reverse: ATGCTCTCAGCCAAGTCTGCCA |
| m-Pfkl | Forward: CCATCAGCAACAATGTGCCTGG |
|  | Reverse: TGAGGCTGACTGCTTGATGCGA |
| m-Pfkm | Forward: CTGTTCGCTCTACCGTGAGGAT |
|  | Reverse: TTGGAACCACCTTGACCAGTCC |
| m-Pfkp | Forward: AAGAGGAAACCAAGCAGTGCGC |
|  | Reverse: TTCCTCGGAGTTTCACGGCTTC |
| r-Ccr7 | Forward: ATGACGTCACCTACAGCCTG |
|  | Reverse: CAGCCCAAGTCCTTGAAGAG |
| r-Cd68 | Forward: CTGTTGCGGAAATACAAGCA |
|  | Reverse: GGCAGCAAGAGAGATTGGTC |
| r-Ccl2 | Forward: GGACCAGAACCAAGTGAGATCAGA |
|  | Reverse: AGCTTCAGATTTATGGGTCAAGTTCAC |
| r-Tnf | Forward: GGATCTCAAAGACAACCAAC |
|  | Reverse: ACAGAGCAATGACTCCAAAG |
| r-Cxcl9 | Forward: GACTCCAGCACGGTGACTTA |
|  | Reverse: ATGCAGGAGCATCGCTGATT |
| r-Ccl3 | Forward: GCTTCTCCTATGGACGGCAA |
|  | Reverse: TGCCGGTTTCTCTTGGTCAG |
| r-Cd86 | Forward: TAGGGATAACCAGGCTCTAC |
|  | Reverse: CGTGGGTGTCTTTTGCTGTA |
| r-Nos2 | Forward: GGATCTTCCCAGGCAACCA |
|  | Reverse: AATCCACAACTCGCTCCAAGATT |
| r-Ccl4 | Forward: CTTCTGCGATTCAGTGCTGTCA |
|  | Reverse: GCAAAGGCTGCTGGTCTCATAGTAA |
| r-Tlr4 | Forward: TCCTTTCCTGCCTGAGACCA |
|  | Reverse: TCAGGGGGTTGAAGCTCAGA |
| r-Ccl5 | Forward: AGGAGTATTTTTACACCAGC |
|  | Reverse: TGACAGGAAAGCTATACAGG |
| r-Rela | Forward: AACACTGCCGAGCTCAAGAT |
|  | Reverse: CATCGGCTTGAGAAAAGGAG |
| r-Il6 | Forward: ACAGTGCATCATCGCTGTTC |
|  | Reverse: CCGGAGAGGAGACTTCACAG |
| r-Ilb | Forward: CTGTGACTCGTGGGATGATG |
|  | Reverse: GGGATTTTGTCGTTGCTTGT |
